# Cogmed Training Does Not Generalize to Real-World Benefits for Adult Hearing Aid Users: Results of a Blinded, Active-Controlled Randomized Trial

**DOI:** 10.1097/AUD.0000000000001096

**Published:** 2021-09-14

**Authors:** Helen Henshaw, Antje Heinrich, Ashana Tittle, Melanie Ferguson

**Affiliations:** 1 National Institute for Health Research (NIHR) Nottingham Biomedical Research Centre, Nottingham, United Kingdom; 2 Hearing Sciences, Mental Health and Clinical Neurosciences, School of Medicine, University of Nottingham, Nottingham, United Kingdom; 3 Manchester Centre for Audiology and Deafness (ManCAD), School of Health Sciences, Faculty of Biology, Medicine and Health, University of Manchester, Manchester, United Kingdom; 4 Nottingham University Hospitals NHS Trust, Derby Road, Nottingham, United Kingdom; 5 Ear Science Institute Australia, Perth, Australia; 6 Faculty of Health Sciences, Curtin University, Perth, Australia.

**Keywords:** Cognition, Hearing aids, Hearing loss, Memory training, Speech perception, Working memory

## Abstract

**Objectives:**

Performance on working memory tasks is positively associated with speech-in-noise perception performance, particularly where auditory inputs are degraded. It is suggested that interventions designed to improve working memory capacity may improve domain-general working memory performance for people with hearing loss, to benefit their real-world listening. We examined whether a 5-week training program that primarily targets the storage component of working memory (Cogmed RM, adaptive) could improve cognition, speech-in-noise perception and self-reported hearing in a randomized controlled trial of adult hearing aid users with mild to moderate hearing loss, compared with an active control (Cogmed RM, nonadaptive) group of adults from the same population.

**Design:**

A preregistered randomized controlled trial of 57 adult hearing aid users (n = 27 experimental, n = 30 active control), recruited from a dedicated database of research volunteers, examined on-task learning and generalized improvements in measures of trained and untrained cognition, untrained speech-in-noise perception and self-reported hearing abilities, pre- to post-training. Participants and the outcome assessor were both blinded to intervention allocation. Retention of training-related improvements was examined at a 6-month follow-up assessment.

**Results:**

Per-protocol analyses showed improvements in trained tasks (Cogmed Index Improvement) that transferred to improvements in a trained working memory task tested outside of the training software (Backward Digit Span) and a small improvement in self-reported hearing ability (Glasgow Hearing Aid Benefit Profile, Initial Disability subscale). Both of these improvements were maintained 6-month post-training. There was no transfer of learning shown to untrained measures of cognition (working memory or attention), speech-in-noise perception, or self-reported hearing in everyday life. An assessment of individual differences showed that participants with better baseline working memory performance achieved greater learning on the trained tasks. Post-training performance for untrained outcomes was largely predicted by individuals’ pretraining performance on those measures.

**Conclusions:**

Despite significant on-task learning, generalized improvements of working memory training in this trial were limited to (a) improvements for a trained working memory task tested outside of the training software and (b) a small improvement in self-reported hearing ability for those in the experimental group, compared with active controls. We found no evidence to suggest that training which primarily targets storage aspects of working memory can result in domain-general improvements that benefit everyday communication for adult hearing aid users. These findings are consistent with a significant body of evidence showing that Cogmed training only improves performance for tasks that resemble Cogmed training. Future research should focus on the benefits of interventions that enhance cognition in the context in which it is employed within everyday communication, such as training that targets dynamic aspects of cognitive control important for successful speech-in-noise perception.

## INTRODUCTION

The ability to hear is central to individuals’ health and wellbeing. Hearing loss is a highly prevalent long-term condition, affecting 1.33 billion individuals worldwide (Vos et al. 2015). It is a leading contributor to years lived with disability (YLD), and the second leading global impairment in 2015 (Vos et al. 2015). In the United Kingdom, approximately 12 million people have a significant hearing loss, which equates to more than one in six of the population, estimated to rise to 1 in 5 people by 2035 (RNID 2015, 2021; Office for National Statistics 2019). The vast majority (92%) of those with hearing loss experience mild to moderate loss.

Sensorineural hearing loss is characterized by declines in both peripheral hearing and central auditory processing, which adversely affect both the audibility and clarity of speech, particularly in noise (e.g., Anderson et al. 2013a; Mattys et al. 2012). Indeed, listening to speech-in-noise is one of the most common complaints of individuals with hearing loss (Kochkin 2002). People with mild and moderate hearing loss face substantial difficulties in communication, which can lead to reduced social participation and quality of life (RNID 2015; Barker et al. 2017; Heffernan et al. 2016). The most common management strategy for hearing loss is the provision and use of hearing aids to amplify sounds (Kiessling et al. 2003). A Cochrane systematic review showed that hearing aids significantly improved listening ability and hearing- and health-related quality of life in adults with mild to moderate hearing loss (Ferguson et al. 2017). Nevertheless, hearing aids cannot restore lost hearing and people with hearing loss often report that even for speech sounds that are loud enough to be heard (i.e., supra-threshold), it is not always clear what is being said. As such, listening can be tiring and effortful (Pichora-Fuller et al. 2016), with listening via hearing aids adding unique cognitive demands (Hafter 2010; Lunner et al. 2009; Lunner & Sundewall-Thorén 2007; Ng et al. 2014; Rudner and Lunner 2013). Hearing loss has been associated with accelerated rates of cognitive decline and an increased risk of developing dementia (Lin et al. 2011b; Lin et al. 2013; Livingston et al. 2017; 2020 Loughrey et al. 2018; Panza et al. 2015; Wayne and Johnsrude 2015), with evidence indicating that hearing loss is the leading modifiable mid-life risk factor for dementia (Livingston et al. 2020). As such, hearing loss is increasingly being recognized as a major public health concern (Livingston et al. 2020; Wilson et al. 2019; World Health Organization 2017, 2018) and interventions to address and modify hearing loss are a priority for health research (Henshaw et al. 2015; Livingston et al. 2020; NICE 2018; World Health Organization 2017, 2018, 2019).

### The Role of Working Memory in Listening

Over the last three decades, there has been growing consensus that factors including cognitive processes are an essential component to listening (an active process), compared with hearing (a largely passive process) (Dryden et al. 2017; Gordon-Salant & Fitzgibbons 1997; Anderson Gosselin et al. 2011; Heinrich et al. 2016a; Heinrich et al. 2015; Heinrich et al. 2016b; Moore et al. 2014; Pichora-Fuller et al. 2016). One of the most frequently cited frameworks to explain auditory-cognitive interactions for speech-in-noise perception is the Ease of Language Understanding Model (ELU; Rönnberg et al. 2019; Rönnberg et al. 2013). The basic assumption of the ELU is that individuals possess a battery of stored phonological representations. For cases where the auditory input fails to match stored phonological representation (for example, where auditory input is degraded as a result of hearing loss, or altered by hearing aid signal processing strategies or background noise), increased demands are placed on working memory to help resolve the mismatch (Classon et al. 2013). Consequently, individuals with greater working memory capacity may be better able to resolve ambiguity, leading to better speech perception in degraded listening conditions (Zekveld et al. 2012).

However, there is a debate in the literature as to whether speech perception relies predominantly on verbal working memory (Rönnberg et al. 2019; Rönnberg et al. 2013), or whether more general working memory, executive, and attentional functions are implicated (see Wayne et al. 2016). Given the widespread difficulties in speech-in-noise perception, and its association with cognition, interventions such as training programs designed to improve speech-in-noise perception or cognition could play an important role in improving everyday communication, social participation and quality of life for people with hearing loss.

### Auditory and Working Memory Training Interventions

Auditory and cognitive training interventions can be delivered via mobile technologies (e.g., smartphones, tablets), computers and the internet, thus offering low-cost forms of self-management support that can be individually tailored and conveniently accessed by people with hearing loss (Ferguson et al. 2015a, 2015b). For any form of training intervention, post-training improvements can be assessed for the trained task(s), termed on-task learning, for untrained tasks, termed off-task learning (or generalization). Generalized improvements can be shown for tasks that are similar to trained tasks (termed near-transfer of learning), or for tasks that are dissimilar to trained tasks (termed far-transfer of learning). In order for a training intervention to be considered effective for people with hearing loss, it should result in sustained generalized improvements that extend beyond trained tasks (i.e. far-transfer of learning), to benefit their everyday listening abilities (Ferguson et al. 2015c).

Auditory training can be broadly described as a process of training the brain to listen through active engagement with sounds (Schow and Nerbonne 2006), and it has been demonstrated to result in improved speech perception over the course of an adult’s lifespan (Wright and Zhang 2009). As such, auditory training can be offered prior to, or alongside hearing aids to help improve outcomes for people with hearing loss (Ferguson et al. 2014; Henshaw & Ferguson 2014). While there is robust evidence to show that auditory training results in improvements for trained tasks, evidence for the transfer of on-task learning to functional improvements for people with hearing loss is mixed (Henshaw et al. 2013a). We suggest two reasons for this. First, published evidence has historically been of very-low to moderate study quality and so we cannot be certain of the reported estimations of effect. Second, the consideration of auditory-only training stimuli may have overly limited the scope of those prior investigations. Indeed, a number of high-quality studies have since been published (e.g., Anderson et al. 2013b; Ferguson et al. 2014; Henshaw and Ferguson 2014; Saunders et al. 2016), which show evidence for auditory training-related improvements in untrained measures of cognition, speech perception and self-reported hearing abilities. Ferguson et al. (2014) were the first to suggest that generalized auditory-training-related improvements may be driven by improvements in cognition (attention and working memory), rather than auditory function. This prompted us to ask the question “could training cognition directly offer a more effective route to real-world benefit?”

Cognitive training is defined as a program of mental exercises designed to maintain or improve core cognitive abilities (Simons et al. 2016). Although the definition of working memory is often debated, most agree that working memory is a flexible, capacity limited, mental workspace used to store and process information in the service of ongoing cognition (see Morrison & Chein 2011). Cognitive training programs that target working memory abilities train the processing, storage, and manipulation of information in order to challenge and improve this system. There are two main approaches to training working memory that differ in terms of their focus on either domain-specific or domain-general components of the working memory system. Domain-specific training targets the development of strategy to enable trainees to recall increasing amounts of information of a particular type (e.g., McNamara & Scott 2001). Domain-general training on the other hand involves core training, such as practice on tasks with increasing working memory demand, to improve domain-general working memory mechanisms (e.g., Klingberg et al. 2002). If successful, core training should result in improvements for tasks that are similar to those trained (near-transfer) as well as improvements for cognitive tasks that are untrained (far-transfer). Furthermore, core training may increase performance for other tasks that are reliant on working memory capacity.

Given the wealth of published evidence for associations between speech-in-noise perception and working memory performance, and in line with predictions from the ELU model, which state that individuals with greater working memory capacity may be better able to resolve ambiguity, leading to better speech perception in degraded listening conditions (Rönnberg 2013; 2019; Zekveld et al. 2012), we aimed to examine whether improving domain-general working memory processes using core training could improve outcomes for adults with hearing loss who use hearing aids. Based on our prior research, which showed significant generalized improvements in working memory, attention and speech-in-noise performance arising from an auditory training task (phoneme discrimination; Ferguson et al. 2014; Henshaw & Ferguson 2014), we sought to identify whether working memory capacity (core training) directly could result in greater transfer of learning to the same outcome measures, both immediately and for a period of time after the training had concluded. The selected training program for the current trial was Cogmed RM, a commercially available working memory training program suitable for use by adults that has been the subject of significant amount of basic and applied research across a range of healthy and clinical populations (e.g., Gathercole et al. 2012; Shipstead et al. 2012, Shinaver et al. 2014).

Cogmed is an 11-task multifaceted training program described as targeting verbal and visuospatial working memory and storage. The majority of training tasks (6/11) target storage aspects of working memory (such as remembering a sequence of numbers, letters, or objects for immediate recall). Some tasks (4/11) required manipulation of information such as recall in reverse or numerical order, and one of the 11 tasks elicits associative memory. Some of the key advantages of Cogmed are that it can be delivered remotely via the internet, making it widely accessible for use at home. Additionally, and importantly for the robust RCT design of the current study, the “research edition” of Cogmed RM offers the ability to effectively blind participants and researchers using adaptive and nonadaptive (placebo) versions of the training. There are however a number of clear limitations to selecting a commercially available training product, including a lack of control over the underlying mechanisms of training, as well as a lack of flexibility in the training protocol itself. This is a particular issue for “kitchen-sink” training programs such as Cogmed that use several different types of task and stimuli designed to impact different components of the working memory system as it becomes difficult to determine which components of the training may underlie subsequent cognitive improvements. However, it is also argued that one advantage of such an approach is that the chance of one, or some combination of, the training tasks might result in beneficial improvements in outcomes for trainees (Morrison & Chein 2011).

The vast literature for the effectiveness of Cogmed training reports mixed findings. Some studies have shown improvements in working memory capacity, individual measures of cognitive control, and fluid intelligence for both adults and children (see Morrison & Chein 2011, Shinaver et al. 2014 for reviews), with improved sustained attention effects reported to persist up to 6 months post-training (Shinaver et al. 2014). Specifically, outcomes domains reported to improve as a result of Cogmed training include cognitive control (Stroop) and fluid intelligence (Raven’s Progressive Matrices) in both healthy young adults and children with ADHD (Klingberg et al. 2002). These findings have been replicated or partially replicated in similar populations (Klingberg et al. 2005; Olesen et al. 2004; Westerberg & Klingberg 2007, Holmes et al. 2009; Holmes et al. 2010) and in studies of healthy children (Thorell et al. 2009). However, many other studies have failed to show transfer beyond trained Cogmed tasks, which has been argued to reflect the components of working memory targeted by this training approach (see Shipstead et al. 2012 for a comprehensive review). Shipstead et al. (2012) argue that greater scientific rigor should be employed across the board, and more attention should be paid to improving component memory processes aligned to the intended target of training (e.g., secondary rather than primary memory for children with ADHD, Gibson et al. 2011). For Cogmed training research in older adults, published studies have examined a fairly restricted set of outcomes, which it has been argued may not be ecologically relevant for older populations (Richmond et al. 2011). At the time of conception of this research, there was some preliminary evidence to suggest that Cogmed training may result in improved sentence repetition skills in a small pilot study of children with profound hearing loss who used cochlear implants (Kronenberger et al. 2011). We sought to expand this field of research to examine applied benefits of Cogmed training to cognition, speech perception, and everyday listening for a population of older adults who used hearing aids, both to generate new knowledge about the associated mechanisms of training and transfer, and to inform future research and intervention development directions.

Here, we report the results of a blinded, randomized active-controlled trial designed to assess the benefits of a 5-week program of Cogmed training for adult hearing aid users aged 50 to 74 years old with mild to moderate hearing loss, compared with an active control group from the same population. In line with published recommendations for high-quality research (e.g., Henshaw & Ferguson 2013a; Jaeggi et al. 2013, Melby-Lervåg et al. 2016), we employ a robust study design, examine individual differences in training and transfer for participants in the Experimental Group, and extend our assessments to examine long-term (6 months post-training) benefits.

#### Primary Hypotheses: Examining the Effectiveness of the Training Intervention Both Immediately and Over Time

1. Hearing aid users in the Experimental Group who receive adaptive Cogmed working memory training will demonstrate significantly improved performance for trained Cogmed tasks (on-task learning) and generalized improvements for untrained measures of cognition, speech perception and self-reported hearing abilities (transfer of learning). Furthermore, these improvements will be significantly greater than for hearing aid users in the active control group who receive nonadaptive training.
2. For participants in the Experimental group, any training-related improvements in untrained outcomes will be maintained 6-months post-training.

#### Secondary Hypotheses: Assessing Individual Differences in On- and Off-Task Learning (for Participants in the Experimental Group Only)

1. On-task learning and post-training performance in untrained outcomes will be predicted by baseline working memory performance.
2. Post-training performance on untrained outcome measures will be predicted by pretraining outcome performance for those measures and the degree of on-task learning achieved during working memory training.

## MATERIALS AND METHODS

This trial is reported in accordance with the CONSORT statement (Schulz et al. 2010). The working memory training intervention elements are reported in accordance with TIDierR guidance (Hoffmann et al. 2014). Approvals were received from the Nottingham Research Ethics Committee 2 (08/H0408/172) and Nottingham University Hospitals Trust Research and Development (08ET002). Research was conducted in accordance with the World Medical Association Declaration of Helsinki. The trial was preregistered (www.clinicaltrials.gov: NCT01892007) and the study protocol prepublished in a peer-reviewed journal (Henshaw et al. 2013b).

### Study Design

A single-center, phase II, active-controlled RCT with participant and outcome assessor blinding, and minimized allocation (Scott et al. 2002) of participants to one of the two groups (adaptive training or nonadaptive training) according to: age (younger = 50 to 62 years/older = 63 to 74 years), sex (m/f), baseline working memory [Backward Digit Span, n trials correct (maximum = 14), low ≤6/high ≥7], and hearing aid use (unilateral/bilateral), with a 1:1 allocation. Group sizes were set with the goal to detect a minimum improvement of 1.5 words (15%) in the primary outcome measure (Visual Letter Monitoring [VLM] Task) based on 80% power and a 1-sided type I error rate of 5%. A pooled SD of 2.118 was used to derive the effect sizes and resulted in a total of 27 participants in each group. With an anticipated participant attrition rate of 15%, a total of 31 participants were recruited per group.

### Participants

Participants were recruited from the NIHR Nottingham Biomedical Research Centre database of hearing research volunteers. Adult hearing aid users aged 50 to 74 years were invited to participate in the study. The age range was selected to make comparisons with prior auditory training studies (Ferguson et al. 2014; Henshaw and Ferguson 2014). All participants reported that they used their hearing aids daily. We also recorded whether participants had received formal musical training, as this has been repeatedly shown to be associated with enhanced speech-in-noise processing (Strait et al. 2012; Varnet et al. 2015).

Individuals who wished to participate were assessed according to the inclusion and exclusion criteria. Eligible individuals took part in an initial screening assessment at the NIHR Nottingham Biomedical Research Centre.

#### Inclusion Criteria

- Adults aged 50 to 74 years old.
- Existing hearing aid users (3+ month’s hearing aid experience, to reduce the likelihood of acclimatization to amplification having an impact on outcome performance).
- Mild to moderate hearing loss [defined as air conduction pure-tone thresholds of 21 to 70 dB HL across octave frequencies (0.25 to 4 kHz) in the better hearing ear, according to Recommended Procedure (British Society of Audiology 2011)].
- Sensorineural hearing loss (defined as an air-bone gap across 0.5, 1, and 2 kHz <15 dB).
- Internet access at home.

#### Exclusion Criteria

- Having previously taken part in a training intervention study
- First language other than English, as speech outcome materials were presented in English
- Unable to use either a desktop or a laptop computer, as training software was accessed via the internet at home
- A score of less than 26/30 on the Montreal Cognitive Assessment, suggestive of mild cognitive impairment (Nasreddine et al. 2005)

### Procedure

**Fig. 1.**
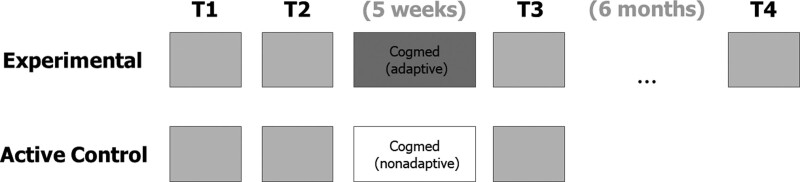
Study design.

Hearing aid users (n = 110) attended an initial screening assessment between September 2012 and March 2014, where informed consent was obtained from those eligible to participate. Following the screening assessment, 62 participants met the inclusion criteria and were recruited into the study. The participants completed two baseline outcome assessments (T1 and T2) at least 2 days apart (mean = 6.9 days, SD = 2.4, range = 2 to 14 days), comprising measures of cognition, speech perception and self-reported hearing (Fig. 1). All outcomes were completed by participants while wearing their hearing aids. Given that performance for the second exposure to a novel measure is assumed to be more reflective of true performance than that of the first exposure, where participants are learning how to complete each task (Ferguson et al. 2014; McArthur 2007), T2 was used as the pretraining baseline. This minimized the risk of procedural learning effects by providing adequate practice and familiarization before the training phase.

On completion of the T2 assessment, participants were randomized into two groups by the lead researcher (H.H.) and allocated log-in details to either the adaptive (experimental group, n = 31) or nonadaptive (active control group, n = 31) web-based working memory training program. Both participants and the researchers conducting the outcome assessments were blind to participants’ group allocation. At the end of the T2 assessment an initial Cogmed familiarization session was conducted by the lead researcher (H.H.), a psychologist who had received appropriate training from, and held a Research Licence Agreement with, Pearson, Inc (the providers of Cogmed training). Following this, the 5-week training program was completed by participants online, in their own homes using their personal computer or laptop, while wearing their hearing aid(s). Training data were automatically uploaded following each session to secure Cogmed servers. Participants were encouraged to contact the research unit if they faced any technical issues with the training program. Progress was monitored remotely by the lead researcher, with telephone calls made to participants if sessions were missed in order to help address any issues.

**Fig. 2.**
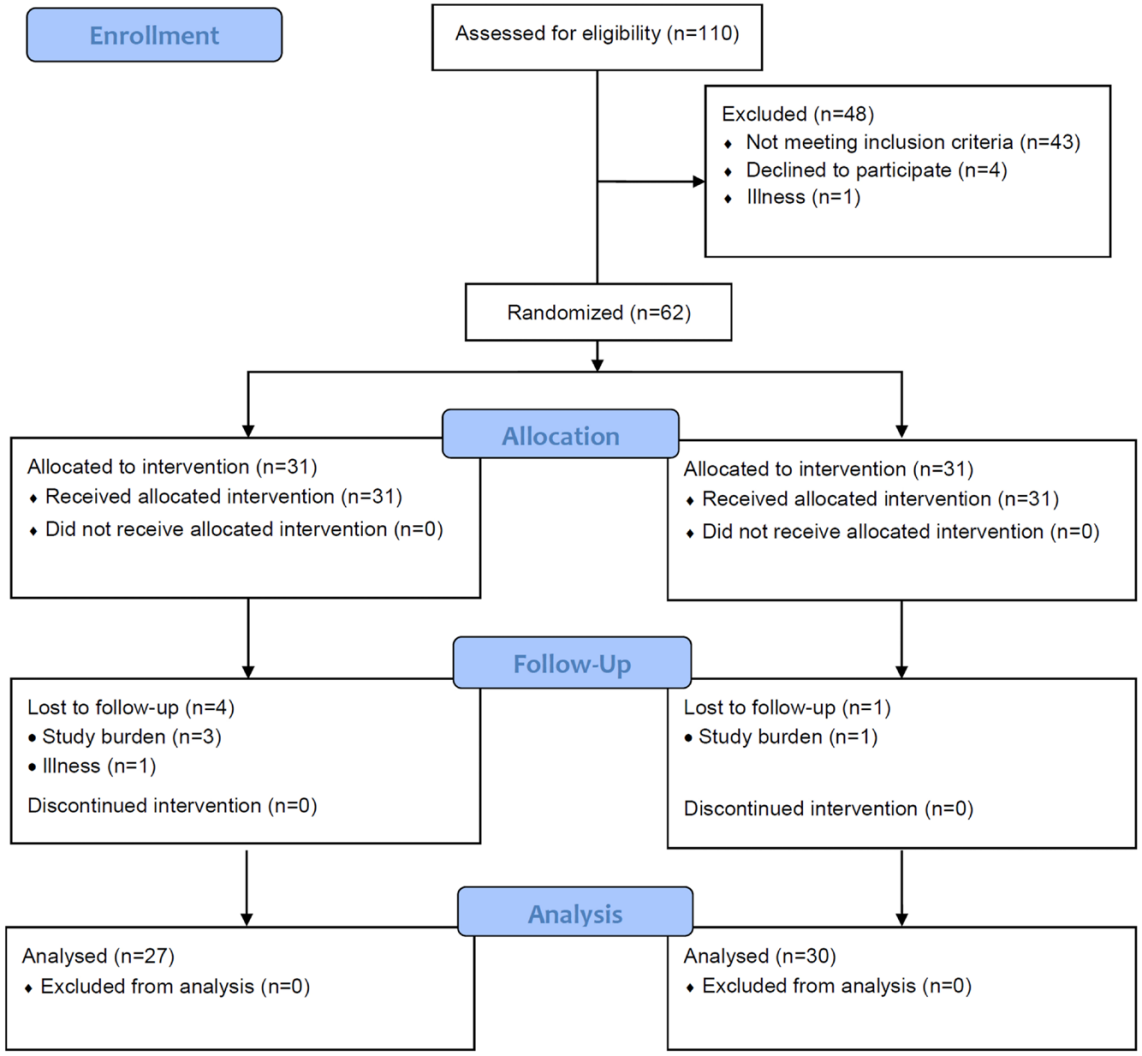
CONSORT flow diagram.

Participants in both groups attended a post-training outcome measures assessment (T3). Those in the Active Control Group completed the trial at this point and were debriefed as to the nature of the trial and were offered the adaptive training to complete at home, if they wished. Those in the Experimental Group only were invited to return to the research unit for a 6-month post-training follow-up appointment (T4). Participants were offered a nominal attendance fee (£5 per hour) and travel expenses for each visit to the research unit, and an inconvenience fee of £20 to, in part, recompense their time for undertaking the at-home training. Five participants (n = 4 Experimental Group, n = 1 Active Control Group) were lost to follow-up at the post-training intervention session T3 (week 7), and five participants in the Experimental Group failed to return for the 6-month follow-up assessment T4 (week 31). Reasons given included illness, the levels of commitment required to complete a longitudinal trial, and the burden of multiple outcome assessments. As per the study protocol (Henshaw and Ferguson 2013b) those participants who withdrew from the study were not replaced. A total of 57 participants completed the trial and their data were included in all subsequent analyses (Fig. 2). Participants in the two groups did not differ significantly in terms of their demographic information (Table 1). Baseline characteristics for the participants who withdrew from the study were statistically comparable to participants who completed their involvement in the research [5 males, 5 females, *x*^2^ (1) = 0.24, *p* = 0.878; mean age = 65.20, SD = 6.46, *t*(65) = −0.164, *p* = 0.870; better-ear pure-tone average audiometric thresholds = 40.80 dB HL, SD = 13.17, *t*(65) = −0.119, *p* = 0.906]; T1 Backward digit span = 7.40, SD = 2.76, *t*(65) = −0.380, *p* = 0.705.

**TABLE 1.** Demographic characteristics and between-group comparisons for participants in the active control (n = 30) and experimental (n = 27) groups

Participant Demographics	Active Control	Experimental	Between-Group Comparison	
	n = 30	n = 27	t/χ^2^	*p*
Age, yr; mean (SD)	63.73 (5.45)	66.22 (6.30)	1.60	0.12
Sex				
Male, n (%)	14 (46.7)	13 (48.1)		
Female, n (%)	16 (53.3)	14 (51.9)	0.01	0.91
Better-ear hearing				
PTA_0.25–4 kHz_ dB HL, mean (SD)	37.53 (12.50)	43.26 (14.48)	1.60	0.12
Montreal Cognitive Assessment score, mean (SD)	28.10 (1.61)	27.93 (1.24)	−0.46	0.65
Baseline working memory				
T1 backward digit span (n trials correct), mean (SD)	7.17 (2.83)	6.96 (2.10)	−0.31	0.76
Hearing aid fitting				
Unilateral, n (%)	2 (6.7)	4 (14.8)		
Bilateral, n (%)	28 (93.7)	23 (85.2)	1.00	0.32
Hearing aid use, yr; mean (SD)	5.14 (7.29)	7.71 (7.81)	1.28	0.21
Musical experience (formal training)				
Yes n (%)	7 (23.3)	4 (14.8)		
No n (%)	23 (76.7)	23 (85.2)	0.66	0.42
Computer skill				
Beginner, n (%)	10 (33.3)	7 (26.9)		
Competent, n (%)	20 (66.7)	19 (73.1)	0.27	0.60

Independent *t* tests used for between-group comparisons for continuous variables. Chi square tests used for between-group comparisons for categorical variables. For definition of computer skill, see Henshaw et al. (2012).

### Audiometric Testing and Cognitive Screening

Audiometric testing took place at the initial screening assessment. Otoscopy was performed according to the BSA recommended procedure for ear examinations (British Society of Audiology 2010). Unaided pure-tone air conduction thresholds at 0.25, 0.5, 1, 2, 3, 4, and 8 kHz were obtained for each ear and pure-tone bone conduction thresholds at 0.5, 1, and 2 kHz as required, following the BSA recommended procedure for pure-tone audiometry (British Society of Audiology 2011). Thresholds were obtained in a sound-attenuated booth using a Siemens Unity PC audiometer, Sennheiser HDA-200 headphones, and B71 Radioear transducer.

The Montreal Cognitive Assessment was administered by a researcher in a quiet testing room. Scores were adjusted according to education level and a score of 26/30 or greater was considered to indicate normal cognitive function (Nasreddine et al. 2005).

### Working Memory Training Intervention

The research edition of Cogmed RM working memory training (Pearson Education, Inc) is an adaptive, web-based training program comprising 11 working memory training tasks, of which participants were required to work through 8 of the 11 possible tasks at each session (as determined by the training software per session, consistent across participants). The training tasks are described as visuospatial and verbal working memory and storage (Henshaw & Ferguson 2013b). Table 2 provides a process-level description of the individual training tasks, which shows that the majority of training tasks target storage aspects of working memory (remembering a sequence of numbers, letters, or objects for immediate recall). Some tasks require manipulation of information such as recall in reverse or numerical order, and one task elicits associative memory. Participants were required to train for approximately 35 to 45 minutes per day, 5 days a week for 5 weeks (total 25 training sessions), completing 8 individual training tasks per day and taking regular breaks where required. Auditory and visual feedback was provided for correct and incorrect responses. Participants completed training at home using their own personal computer, laptop or tablet PC.

**TABLE 2.** Task description and details of the targeted memory process for each of the 11 individual Cogmed training tasks

Task Name	Marketed Memory Process	Type of Recall	Storage and/or Manipulation	Notes
Description provided by Cogmed				
**3D Cube**				
A number of panels will light up in different colors in succession. At the same time the cube is turning towards each panel that lights up. The user needs to remember the order in which they lit up. When it says “go”, the user clicks on the panels in the same order.	Visuospatial WM	Serial order	Storage	Taxes only storage.
**Asteroids**				
A number of moving asteroids will light up in succession. The User needs to remember the order in which the shapes lit up. When it says “go”, the User clicks on the shapes in the order they lit up.	Visuospatial WM	Serial order	Storage	Taxes only storage.
**Data Room**				
Some of the lamps in a 3D room will light up. The User needs to remember the order, and then click on the lamps in the order that they lit up.	Visuospatial WM	Serial order	Storage	Taxes only storage.
**Decoder**				
Certain letters will be said aloud. At the same time, the letters will light up. The User needs to remember the letters that he/she hears and select the letters by clicking on them. Example: The following letters will be heard: “D, P, E.” The first letter is ‘D”—you have to select that letter from the three options under the first lamp. At the next lamp, you must select ‘P’, the second letter. Finally, you must select the ‘E’ from the choices under the third lamp.	Verbal and visuospatial span	Serial order	Storage	Verbal aspects of this task are limited to the recall of spoken letters in the order in which they were presented.
**Input module**				
A number of digits will be read out loud in succession. The User needs to listen carefully and try to remember the order in which they were read. When it says, “go”, the participant clicks on the numbered buttons in the reverse order.	Verbal and visuospatial WM	Reverse order	Storage and manipulation	Verbal aspects of this memory task are limited to the recall of digits that are both spoken and visually presented, in reverse order.
**Input module with lid**				
This is a different version of the Input module exercise. The numbers are read aloud; however, the user cannot see the numbered buttons as they are read. the numbers will appear when it is the user's turn to click on the numbered buttons in reverse order.	Verbal WM	Reverse order	Storage and manipulation	The verbal aspects of this memory task are limited to the recall of spoken digits in reverse order.
**Rotating dots**				
The user will see some lamps rotating. the lamps will light up in a certain order. the lamps will also move so the user needs to keep track of their position. the user then clicks on the lamps in the same order, even though they are now in new positions.	Visuospatial WM	Serial order	Storage and manipulation	Visual tracking of rotation required to accurately report the order of the lamps that light up in the same order as they were presented.
**Sorter**				
Certain boxes will be highlighted and numbers will be revealed. They will then disappear. When it says “go”, the User begins by clicking on the box that contains the number 1, then the box that contains the number 2, 3, and so on, in numerical order.	Visuospatial WM	Numerical order	Storage and manipulation	Accurate responses require the storage of visually presented number locations and their recall in numerical order.
**Space Whack**				
Monsters will randomly appear in craters. Before they come out, they let out a little cloud of gas and the user needs to remember the pattern of the gas clouds in order to be able to hit the monsters on the head when they appear. It is important that the user waits until all gas clouds have shown and is prepared to hit each monster by starting with the pointer above the first crater.	Visuospatial WM	Serial order	Storage	Taxes only storage.
**Stabilizer**				
Certain letters will be read aloud. When a letter is read, it will be displayed in the middle of the circle, and at the same time, a corresponding light will light up. After all the letters have been read, one of them will be displayed once again in the middle. The user needs to remember which light came on when he/she heard that particular letter. The user answers by clicking on the correct light.	Verbal and visuospatial WM	Associative	Storage and manipulation	Verbal aspects of this memory task are limited to the recall of letters that are both spoken and visually presented. Accurate responses require the storage and recall of formed stimuli associations.
**Visual data link**				
A number of lamps will light up in succession. The user needs to remember the order in which they came on. When the program says, “go”, the user must click on the lamps in the same order that they lit up.	Visuospatial WM	Serial order	Storage	Taxes only storage.

#### Adaptive Training

Individuals randomized to the Experimental Group received an adaptive version of Cogmed RM working memory training, where training task difficulty (number of to-be-remembered items) was varied based on individual performance, to maintain average daily levels of 60% trials correct.

#### Nonadaptive Training

Individuals randomized to the Active Control Group received identical training software and training protocols. However, training task difficulty was fixed at 3 to-be-remembered items and did not adapt with individual performance.

#### Training Aides

Wherever possible, participants were supported by their most frequent communication partner (typically their spouse), who served as “Training Aide”. This is a requirement of Cogmed training and serves to monitor progress, avoid undesired tactics (such as writing down numbers or saying numbers out loud), offer encouragement, and suggest rest-breaks should participants become tired or frustrated with training tasks. This was particularly important for individuals in the Experimental Group who received a challenging adaptive training program, but was implemented in the same way across both groups to facilitate participant and researcher blinding. Training aides supported 28/30 (93.3%) of participants in the Active Control Group and 24/27 (88.9%) of participants in the Experimental Group. Training aides attended the unit with participants at the pretraining assessment (T2) where they were briefed on their role. Both participants and training aides were introduced to the training software via a short demonstration at the end of the assessment session. The demonstration was the same for both participant groups.

#### Time-on-Task Weighting

Given that the Active Control Group had a task that inherently required less time to complete than the Experimental Group (less to-be-remembered items resulting in shorter trials), the number of trials overall was weighted (increased by 30%, the maximum available weighting allowed by the training software) to help equate the total time spent training between the two groups.

### Cognitive and Speech Testing

Cognitive and speech perception measures were completed in a purpose-designed quiet test room. Visual stimuli were presented using a 21″ screen (Genelec, Inc) placed 50 cm in front of the participant. Unless otherwise stated, auditory stimuli were delivered via a Logitech LS11 speaker placed directly in front of the participant at a distance of 1 m. Participants wore their hearing aid(s) throughout testing. Cognitive and speech perception measures were obtained in a fixed order that was the same for all participants and all assessment sessions. Volume levels were set to the individual participants most comfortable loudness level (most comfortable loudness [MCL]) (Ventry, Woods, Rubin, & Hill 1971) at the first outcome assessment session (T1) and recorded and maintained for each of their subsequent assessments. To determine individuals’ MCL, a list of 5 AB words in quiet (Boothroyd 1968) were played on continuous loop with the speaker volume turned down and participants were asked to indicate to the researcher when the speaker volume was loud enough so that they could clearly hear all of the words but not uncomfortably loud. To ensure the MCL level was appropriate, participants were given a second list of AB words in quiet at their chosen MCL and were asked to repeat them back. If any of the five words were repeated incorrectly, or if participants’ were unhappy with the MCL they had set, step 1 was repeated.

### Outcome Measures

Measures were selected to assess improvements for trained tasks, domain-general improvements in working memory, and any associated improvements in outcome domains important to people with hearing loss and hearing aid users (cognition, speech perception and self-reported hearing, Ferguson et al. 2014; Granberg et al. 2014).

#### Primary Outcome Measure •

##### Visual Letter Monitoring task (VLM), Working Memory Updating (Untrained) •

The VLM task is a visual task of working memory updating (Gatehouse, Naylor, & Elberling 2006), which is not trained within the Cogmed working memory training program. Ten consonant-vowel-consonant (CVC) words are embedded in an 80-letter sequence. Two sequences are presented to participants at each outcome assessment in a counterbalanced order, at varying difficulty levels. Individual letters are displayed sequentially on a computer screen at a rate of 2 seconds per letter (least difficult condition: VLM 2 seconds/letter) and 1 second per letter (most difficult condition: VLM 1 second/letter). Participants are asked to press the keyboard “space bar” (hit) when three consecutive letters formed a recognized CVC word (for example, M-A-T). Task performance is scored as the total number of hits (maximum score of ten per list). VLM was selected as the primary outcome measure as it provides an opportunity to examine transfer of learning from trained Cogmed tasks to an untrained working memory task, thus assessing domain-general improvements in working memory capacity. The use of VLM also enables us to draw direct comparisons between results in this trial with our prior studies of auditory training interventions in similar populations (Ferguson et al. 2014; Henshaw and Ferguson 2014).

#### Secondary Outcome Measures •

##### Cognition •

###### Backward Digit Span, Simple Span Working Memory (Storage and Manipulation, Trained) •

The backward digit span, which is trained within Cogmed (two training tasks; Input module, Input module with lid) is assessed outside of the training software as a separate task within the outcome measure battery. A subtest from the Wechsler Adult Intelligence Scale, Third Edition (WAIS-III) (Wechsler 1997), this task involves listening to a string of numbers of increasing length and repeating them in reverse order. The test is presented using prerecorded digits delivered using the Medical Research Council (MRC) Institute of Hearing Research System for Testing Auditory Responses (IHR-STAR) platform via a speaker situated directly in front of the participant. Trials begin with strings of two numbers, finishing at strings of eight. Each string length is presented twice. Strings increased in length by one digit if participants correctly recall one of the two digit strings at each length, otherwise the test is discontinued. Task performance is scored as the total number of strings correctly recalled in reverse order (of a maximum 14 trials).

###### Size-Comparison Span; Complex Span Interpolated Working Memory (Storage, Manipulation and Inhibition, Untrained) •

The Size-Comparison Span (SICSPAN) is a measure of complex-span working memory capacity and inhibition of semantic current-list intrusions (Sorqvist, Ljungberg, & Ljung 2010). The nature of the task closely resembles the “operation span” (OSPAN) task (Engle 2002), with an important addition of measuring intrusions from items that are part of the task itself, but were never intended for recall. Participants view lists of size comparisons (e.g., “tree is larger than acorn”) and respond “yes” or “no” using a button box. Participants are then provided with to-be-remembered words from the same semantic category (e.g., “leaf”). At the end of the list, participants are required to recall the to-be-remembered words, while inhibiting words included in the size comparison judgments. The task begins with lists of two size comparison judgments and to-be-remembered words, increasing to list lengths of three, four, five, and six. There were two trials at each list length. The task continues until all list lengths have been presented, with no discontinuation rule. The task is scored using the number of list items correctly recalled (maximum 40). A second measure (intrusions) records the number of incorrect items recalled (errors in inhibition) for each participant. The SICspan serves to assess domain-general transfer of learning in terms of increased performance on a complex-span working memory task and improved attentional control, both of which are reliant on working memory capacity (see Tiego et al. 2018 for a discussion).

###### Dual Task of Listening and Working Memory; Complex Span Working Memory (Storage and Task Switching, Untrained) •

The dual task is measure of listening and memory designed to index listening effort (Howard, Munro, & Plack 2010). Participants are presented with a five-digit memory task that flanks a speech-in-noise comprehension task. A string of five digits is displayed visually on a computer screen for 5 seconds. Participants are asked to retain the digits in memory for later recall. Participants are then presented with a list of 5 AB isophonemic monosyllabic CVC words (Boothroyd 1968), such as ship, rug and rail, presented in (ICRA) 6-talker babble (Dreschler et al. 2001) at challenging signal-to-noise ratio [0 dB signal-to-noise ratio (SNR); Henshaw & Ferugson 2014] and are asked to repeat each word immediately after presentation. After each list of five words, participants are asked to recall the previously presented five digits. There are 4 word lists, resulting in a maximum possible score of 20 correctly repeated words and 20 correctly recalled digits. A dual task score is calculated by adding together the scores for the word and digit tasks (maximum 40). Prior research has shown improved dual task performance in adult hearing aid users following an average of ~3.25 hours of phoneme discrimination training (Henshaw & Ferguson 2014). This task serves to assess domain-general improvements in working memory arising from improved working memory capacity.

###### Test of Attention in Listening; Sustained Selective Attention (Untrained) •

The Test of Attention in Listening (TAIL) is an auditory attention task that requires participants to make same/different judgements to serially presented tones that vary in both frequency and spatial location (Zhang, Barry, Moore, & Amitay 2012). Participants are asked to respond as to whether two tones are the “same” or “different” in terms of either frequency or location using a button box response. Tones are presented in the freefield at participants’ most comfortable loudness (MCL) level (Ventry et al. 1971) via two speakers situated at 90° to the left and 90° to the right of the participant at a distance of 50 cm. Tone frequencies are drawn randomly from the range 476 to 2000 Hz, with the constraint that the spectral gap between any two tones is at least 2.1 equivalent rectangular bandwidths (Zhang, Barry, Moore, & Amitay 2012). An upper frequency limit of 2000 Hz was selected to help ensure that tones were audible by participants with high-frequency hearing loss. TAIL measures the ability to focus selectively on a task-relevant dimension (either frequency or location) and ignore information from task-irrelevant dimensions, using reaction time (RT) as the primary performance measure. This test produces two measures per condition (frequency and location), calculated from the RT data; Distraction and Conflict Resolution. Distraction measures the RT cost of involuntary orientation to the task-irrelevant dimension in terms of processing efficiency, whereas Conflict Resolution measures the RT cost of resolving that conflict (and suggests the involvement of executive control to help resolve) (Zhang, Barry, Moore, & Amitay 2012). This task serves to assess improvements in an auditory task of sustained attentional control, which is the ability to maintain goal-relevant information in the face of distraction, resulting from any improvements in working memory capacity (e.g., Kane & Engle 2003. See also consensus paper (preprint) by von Bastian et al. 2020).

###### Test of Everyday Attention, Single and Dual Attention (Untrained) •

Test of Everyday Attention (TEA) subtests 6 (telephone search) and 7 (telephone search while counting) assess single (visual) attention and dual (auditory and visual) attention (Robertson, Ward, Ridgeway, & Nimmo-Smith 1994). For subtest 6, participants are asked to search a telephone directory for matching symbols. For subtest 7, participants are asked to search a telephone directory for matching symbols while counting strings of beeps in varying lengths (2 to 12), presented in the freefield at participants’ MCL level, then reporting back the total number of beeps. The task is scored using time (seconds) per correctly identified symbol, weighted in subtest 7 by the proportion of correctly counted beep strings. A dual task decrement is calculated as the difference in time (in seconds) per correctly identified symbol where two simultaneous tasks are being completed, compared with that for the single task (subtest 7 minus subtest 6). Improvements in the dual task decrement (i.e., the ability to divide attention efficiently) would be indicative of improved executive attention, which is closely related to both working memory capacity and executive function (McCabe et al. 2010).

##### Speech Perception •

Measures of speech perception performance were selected to assess generalized benefits to speech perception on a continuum of degree of association with working memory (Heinrich et al. 2015), ranging from speech constituents in quiet (phonemes, low), target words in high and low predictability sentences in babble (mixed), to competing speech (high).

###### Phoneme Discrimination •

This speech perception task assesses an individual’s ability to distinguish differences between phonemes presented on a continuum. The measure is delivered using the Medical Research Council (MRC) Institute of Hearing Research System for Testing Auditory Responses (IHR-STAR) platform. Participants are presented with three discrete phonemes per trial from a continuum of 96 sound files (48 for each phoneme within a pair), which are digitally synthesized from recorded phoneme endpoints. For each trial, two of the phonemes are identical and one is different. Participants are asked to identify the odd one out using a button box with three buttons corresponding to the visual display. Two different phoneme pairs: /a/ /e/ (easy) and /d/ /g/ (difficult) are presented for a block of 35 trials in sequential blocks, with a 3-trial demonstration of continuum /a/ /e/ prior to the 2 blocks. The task is presented in a quiet room, and task difficulty is adapted based on individual participant performance using a three-phase adaptive staircase procedure (Moore et al. 2005). Auditory and visual performance feedback is provided to participants after each trial (correct/incorrect response). This task provides a phoneme discrimination threshold, which is calculated as the average distance between the 96 sound files for the last 2 reversals in a block of 35 trials.

###### British English Semantic Sentence Test •

Previously described as the IHR-SPIN (Henshaw and Ferguson 2013b), and based on the Revised Speech Perception in Noise Test (Bilger et al. 1984; Kalikow et al. 1977), the British English Semantic Sentence Test is a high- and low-predictability speech in noise perception test with sentences produced by a British English native speaker. Lists of 22 sentences (11 high-predictability and 11 low-predictability) are presented in the freefield in a background of speech-modulated noise with the same long-term average spectrum as the target speech (Knight & Heinrich 2017) at a fixed SNR of −1 dB SNR. Two practice sentences (one high- and one low-predictability) are presented to participants at a slightly more favorable fixed SNR (2 dB SNR), prior to commencing the main test. Participants are asked to listen to each sentence and repeat the last word aloud. The task is scored as the percentage of last words correctly repeated for both high-predictability and low-predictability lists.

###### Modified Coordinate Response Measure •

The Modified Coordinate Response Measure is measure of target talker speech perception in the presence of another talker, presented at an adaptive signal-to-noise ratio. The basic task is described by Hazan et al. (2009) and is based on the Coordinate Response Measure (Bolia et al. 2000). Participants are presented with sentences in the form of “show the [animal] where the [color] [number] is”. There are six possible monosyllabic animals (cat, cow, dog, duck, pig, and sheep), six colors (black, blue, green, pink, red, and white), and eight numbers (one to nine, excluding multisyllabic seven). Two sentences are presented to participants concurrently, one produced by a female speaker (target) and one by a male speaker (distracter). Participants are asked to listen for the color and number spoken by the female speaker (“dog” was always the animal target), while ignoring the male speaker, and then respond by pressing the corresponding target color number on a touchscreen computer. The test uses an adaptive 1-up 1-down staircase method with an initial step size of 10 dB until the first reversal, reducing to 7 dB at reversal 2 and 4 dB at reversal 3. The test continues until a total of eight reversals are achieved. The test is completed twice by each participant and a third time for instances where there is a difference of ≥5 dB SNR between participants’ first two test scores. Speech reception thresholds are calculated using the average of the last two reversals, averaged across the 2 or 3 trials.

##### Self-Reported Hearing •

###### Glasgow Hearing Aid Benefit Profile •

The Glasgow Hearing Aid Benefit Profile (GHABP) is a validated questionnaire used to assess self-reported activity limitations (Initial Disability) and participation restrictions (Handicap) arising from difficulties in hearing, as well as hearing aid use, benefit, and satisfaction (Gatehouse 1999). For the purposes of the present study only the first two measures (Initial Disability and Handicap) were assessed in order to make comparisons with prior auditory training studies (Ferguson et al. 2014; Henshaw and Ferguson 2014). The questionnaire is administered via interview with the researcher and completed electronically. Participants are presented with a series of four listening scenarios (listening to the television, having a conversation with one other person in a quiet room, having a conversation in a busy street or shop, talking to several people in a group) and are asked to rate the amount of difficulty they have in that situation while wearing their hearing aid(s) (1 = no difficulty to 5 = cannot manage at all), together with how much any difficulty either worried, annoyed or upset them (1 = not at all to 5 = very much indeed). The mean of all four scenarios in each measure are converted to a percentage score for the Initial Disability and Handicap subscales.

###### Hearing Handicap Inventory for the Elderly •

The Hearing Handicap Inventory for the Elderly is a 25-item validated questionnaire that quantifies the emotional and social/situational effects of self-perceived hearing impairment to quantify hearing-related quality of life. Participants were asked to complete the 25-item paper questionnaire answering statements such as “Does a hearing problem cause you to be nervous” with either “yes” (4 points), “no” (0 points), or “sometimes” (2 points). The questionnaire was scored as total points for all items (maximum 100 points). Subtotal scores can also be derived for emotional (12 items, maximum 48 points) and situational item subscales (13 items, maximum 52 points).

### Statistical Analyses

#### Participant Demographics and Baseline Comparisons

Demographic information was presented for individuals in the Experimental and Active Control Groups as the mean and SD for continuous data, or number and percentage for categorical data. Baseline comparisons assessed whether Experimental and Active Control Groups were comparable in terms of demographics at the outset. Significant differences between groups were assessed using either independent *t*-tests or Chi square.

#### Primary Analyses

Primary analyses assessed on-task learning, transfer to improvements in performance for untrained outcome measures of cognition, speech perception, self-report (Hypothesis 1), and retention of outcome improvements at the 6-month follow-up assessment (Hypothesis 2). Retention of learning was defined as the maintenance of any significant improvement from baseline (T2) at the 6-month follow-up assessment (T4), rather than a nonsignificant decline in performance post-training (T3–T4). This was because we were particularly interested (given the applied potential of training as a supplementary intervention) in examining whether any training-related improvement from baseline outcome performance could persist for a period of time after the training had ceased.

For all primary analyses, missing data were assumed to be missing at random and were handled as follows. For instances where, for a particular variable, data from less than three individuals were missing, plausible values were imputed on an individual basis and complete case analyses were run to assess the effect of imputation on statistical group results. Imputed values were determined by one of the following methods: when the data point for T2 was missing but T1 present, the T1 data point was substituted as the most realistic value. If the data point was missing from T3 or T4, the mean of the individual’s remaining data points (T2 and T3 for missing T4, or T2 and T4 for missing T3) was calculated and imputed as most plausible value. In these cases, checks were conducted to ensure that the selected imputation method did not change the qualitative nature of the group results (turning a result from nonsignificant to significant or vice versa) by running a complete case analysis (Jakobsen and colleagues 2017), whereby missing values were substituted by the minimum and maximum values for the variable from the group as a whole in order to test the statistical robustness of these boundary conditions. Results of all imputation sensitivity analyses are presented in Table 1 in Supplemental Digital Content 1, http://links.lww.com/EANDH/A855. Given that boundary scores did not change the qualitative nature of the statistical outcome, all reported results are based on the above described imputation of realistic values. Finally, for cases where data from three or more individuals was missing for a particular variable, data were imputed using Multiple Imputation by Chained Equation (van Buuren & Groothuis-Oudshoorn 2011). Five imputation cycles were used, as this has previously been demonstrated to produce acceptable results (van Buuren et al. 1999).

##### Working Memory Training (On-Task Learning) •

On-task learning was assessed using the Cogmed Index Improvement. This cumulative performance measure was calculated using the Start Index (average performance across all training tasks completed during training sessions 2 and 3) subtracted from the Maximum Index (average performance for the two training sessions with the best performance across the full training period). On-task learning was assessed within and between groups for the training intervention period (weeks 3 to 7) using paired and independent *t*-tests.

##### Transfer of Learning to Untrained Outcomes •

The primary endpoint for the trial was T3 (week 7). Generalized Estimating Equations (GEE) were used to examine performance on untrained measures of cognition, speech perception and self-reported hearing as a function of time (T2–T3) between treatment groups; hence the effect of interest is an interaction between group (control versus treatment) and time (T2 versus T3). As only the interaction between group (experimental versus control) and visit (T2 versus T3) was of interest, no post-hoc analyses were required. Change in performance pretraining to post-training (T2-T3) for both treatment groups are presented with 95% confidence intervals.

We initially planned to use Repeated Measures ANOVA to assess transfer of learning (Henshaw & Ferguson 2013b). However, following subsequent advice of a medical statistician who had experience in analyzing repeated measures data, we adopted GEEs as a more rigorous analysis that is better suited to datasets where outcome measures are correlated across time points and within participants (Zeger, Liang, & Albert 1988).

##### Retention at Follow-Up •

GEEs were used to examine performance across T2 to T4 for the Experimental Group only in order to examine retention of any post-training improvements at a 6-month post-training follow-up assessment. Posthoc comparisons between T2 to T3 and T2 to T4 were performed using unadjusted *t*-tests.

For on-task learning, effect sizes were calculated using Glass’s delta (Hedges & Olkin 1985), which is suited to between-group comparisons with unequal variance, and uses the SD of the control group to estimate the size of effect (e.g., Glass’s delta of 1.5 indicates that the mean of the training group is 1.5 SDs higher than the mean of the trained group). For all other analyses, effect sizes were calculated using Cohen’s *d* (Cohen 1988), with small, medium and large effects interpreted as 0.2, 0.5, and 0.8 respectively.

#### Secondary Analyses

Secondary analyses assessed individual differences in the transfer of learning to untrained outcomes. As such, all secondary analyses were completed using observed data only (without imputation).

For participants in the Experimental Group only, regression models were used to examine whether:

1. Individuals’ on-task learning and degree of improvement pre- to post-training (T2 to T3) for untrained outcomes could be predicted by their baseline working memory performance (T2 Backward Digit Span) (Hypothesis 3).
2. Individuals’ post-training (T3) performance on untrained outcome measures could be predicted by their pretraining (T2) outcome performance for those measures and the degree of on-task learning achieved during working memory training (Hypothesis 4).

For (a), regression models were used to assess the predictive value of individuals’ baseline working memory performance (Backward Digit Span T2, n trials correct) on their on-task learning (Cogmed Index Improvement) and post training (T3) performance in untrained outcomes of cognition, speech perception, and self-reported hearing.

For (b), stepwise forward regression models (Enter method) were conducted to assess the predictive value of 1. individuals’ baseline outcome measure performance (T2), 2. the degree of on-task learning (Cogmed Index Improvement), and 3. any interaction between the two, on post-training performance (T3) for each outcome measure individually.

For primary and secondary analyses, statistical significance was set to *p* < 0.05 (two-sided), unless otherwise stated. GEE Analyses were conducted using R version 3.3.3 (R Core Team 2017) and packages “geepack” (Højsgaard et al. 2006), “mice” (van Buuren and Groothuis-Oudshoorn 2011), “dplyr” (Wickham et al. 2017), and “compute.es” (Del Re 2013). IBM SPSS Statistics 24 (IBM Corp. 2016) was used for all other analyses.

#### Quality Assurance

Test-retest reliability, standard error of measurement (SEM), and the minimal difference (MD) required to determine true intervention effects was estimated for all outcome measures based on the data from T1 and T2. Test-retest reliability, which indicates the relative consistency of scores, was calculated using intra-class correlation coefficients (ICC) following the procedure of Weir (2005). The choice of ICC was determined by whether or not a significant group difference of repeated testing in an outcome variable existed between T1 and T2. When no significant mean difference of repeated testing existed, ICC3,1 was used, otherwise ICC2,1 was selected. The SEM indicates the absolute precision of a score (Hopkins 2000; Weir 2005). Finally, the MD was calculated to define the smallest difference/change in outcome measure performance that can be considered to represent a “real” post-training difference. For a more detailed discussion of the choice of ICC, SEM, and MD in relation to hearing-related measures, see also Heinrich et al. (2019) and Heinrich & Knight (2020).

## RESULTS

### Primary Analyses

#### Working Memory Training: On-Task Learning (Hypothesis

All of the 57 participants completed 25/25 training sessions (100% adherence). Mean data for Cogmed measures by group, and statistical comparisons between groups, are presented in Table 3.

**TABLE 3.** Cogmed working memory measures for participants in active control (n = 30) and experimental (n = 27) groups

Cogmed Measure	Active Control	Experimental	Between-Group Comparison			
	n = 30	n = 27	df	t/Z	*p*	Effect Size
Start index; mean (SD, range)	39.04 (3.85, 29.38–40.53)	78.95 (6.79, 65.30–92.54)	55	−6.80	<0.001*	10.37‡
Maximum index; mean (SD, range)	39.16 (3.55, 29.38–40.53)	103.43 (10.67, 86.41–126.88)	55	−6.85	<0.001*	18.10‡
Active training time per session (min)	29.89 (4.92, 17.94–42.34)	40.87 (3.72, 34.40–51.21)	55	9.43	<0.001†	2.52§

*Mann-Whitney *U* test.

†Student’s *t* test.

‡Glass’s Δ.

§Cohen’s d.

The average Cogmed Index Improvement for adults aged 50 to 74 years old in the Experimental Group was 24.48 (SD = 6.53, range = 9.74 to 38.84), which is slightly lower than the published mean Cogmed Index Improvement of 30 for adults aged 18 to 65 years old (Cogmed Working Memory Training 2011). Participants in the Active Control Group were unable to improve as their training tasks were presented at a fixed difficulty level of 3 to-be-remembered items, with no adaptation. For participants in the Experimental Group, a comparison of the Cogmed Start Index (mean = 78.95, SD = 6.79) and Maximum Index (mean = 103.43, SD = 10.67) showed a significant on-task learning effect (*t*[26] = −19.468, *p* < 0.001).

Despite best efforts to weight the Active Control Group for time-on-task using the weighting function inherent within Cogmed (maximum applied), the average time-on-task for the Experimental Group was significantly different between groups. On average, individuals in the Experimental Group trained for approximately 11 minutes longer per session, compared with individuals in the Active Control Group (mean difference = 10.99, Cohen’s *d* = 2.52). Nevertheless, a linear regression analysis controlling for time-on-task showed that training group remained a highly significant predictor of the Index Improvement score, *F*(2, 54) = 207.091, *p* < 0.001.

#### Generalization of Learning to Improvements in Untrained Outcomes (Hypothesis 1)

Table 4 shows group means and standard deviation for all untrained outcomes at all measurement time points (T1 to T3 for the Active Control Group; T1 to T4 for the Experimental Group). Missing data were imputed as outlined in the methods, and did not exceed 5.3% for any of the outcome measures across all participants at the pretraining baseline (T2), or the primary endpoint (T3).

**TABLE 4.** Mean and SD performance data for all outcome measures across all time points for participants in the active control (n = 30, T1–T3) and experimental (n = 27, T1–T4) Groups

Outcomes	Active Control						Experimental							
	n = 30						n = 27							
	T1		T2		T3		T1		T2		T3		T4	
	N	Mean (SD)	N	Mean (SD)	n	Mean (SD)	n	Mean (SD)	n	Mean (SD)	n	Mean (SD)	n	Mean (SD)
Cognition														
Visual letter monitoring														
Hits, 2s/letter	30	8.27 (1.89)	30	8.73 (1.48)	30	8.93 (1.55)	27	8.48 (1.42)	27	8.07 (2.59)	27	8.33 (2.04)	24	8.63 (1.97)
Hits, 1s/letter	30	6.67 (2.50)	30	6.93 (2.56)	30	7.07 (2.73)	27	5.74 (2.36)	27	5.93 (2.95)	26	6.73 (2.01)	24	6.50 (2.25)
Backward Digit Span														
Trials correct	30	7.17 (2.83)	30	8.00 (3.06)	30	7.83 (2.68)	27	6.96 (2.10)	26	7.69 (1.78)	25	8.70 (2.19)	22	8.82 (2.36)
Size Comparison Span														
Total	30	24.40 (7.82)	30	26.27 (8.31)	30	26.43 (8.16)	27	20.96 (6.16)	27	24.26 (7.02)	27	25.11 (7.05)	22	24.64 (6.07)
Intrusions	30	3.20 (2.33	30	3.97 (3.35)	30	4.10 (3.68)	27	4.82 (3.63)	27	5.11 (4.10)	27	4.52 (4.01)	22	5.59 (4.15)
Dual task listening and memory														
Dual task score	30	18.87 (5.14)	30	20.20 (6.20)	28	22.14 (5.53)	27	18.11 (6.66)	27	19.26 (5.77)	26	20.88 (5.73)	21	20.71 (5.14)
Test of Everyday Attention														
Subtest 6	30	3.35 (0.65)	30	3.23 (0.70)	30	3.00 (0.69)	27	4.11 (3.67)	27	3.27 (0.63)	27	3.25 (0.66)	24	3.09 (0.55)
Subtest 7 dual task decrement	30	1.26 (1.48)	30	1.26 (2.98)	29	0.55 (0.78)	27	0.19 (3.03)	27	1.11 (1.95)	27	0.80 (1.06)	24	0.81 (0.92)
Test of Attention in Listening														
Attend frequency IO	30	0.04 (0.11)	30	0.07 (0.13)	30	0.06 (0.10)	26	0.06 (0.15)	27	0.01 (0.13)	26	0.03 (0.14)	24	0.06 (0.11)
Attend frequency CR	30	0.02 (0.11)	29	0.05 (0.10)	30	0.05 (0.12)	26	−0.02 (0.09)	27	0.02 (0.11)	26	0.01 (0.10)	24	0.05 (0.09)
Attend location IO	30	0.11 (0.13)	30	0.08 (0.11)	30	0.09 (0.10)	26	0.11 (0.11)	27	0.11 (0.13)	26	0.10 (0.14)	24	0.12 (0.15)
Attend location CR	30	0.10 (0.15)	30	0.10 (0.10)	30	0.10 (0.08)	26	0.10 (0.12)	27	0.11 (0.13)	26	0.14 (0.12)	23	0.15 (0.17)
Speech perception														
Phoneme discrimination														
Threshold/a/ /e/	29	73.79 (13.58)	30	68.47 (11.93)	30	65.20 (9.58)	27	69.98 (8.97)	25	65.92 (7.39)	27	69.59 (11.67)	24	64.96 (8.39)
Threshold/d/ /g/	29	89.88 (10.18)	30	87.07 (12.65)	30	88.40 (12.09)	27	86.44 (11.26)	25	89.10 (11.46)	27	90.07 (11.00)	25	85.67 (13.63)
British English Semantic Sentence Test														
High context (%)	29	33.23 (26.07)	29	24.16 (17.90)	29	29.48 (21.12)	25	31.65 (23.92)	25	30.18 (18.47)	27	29.98 (23.22)	20	22.73 (21.77)
Low context (%)	29	14.75 (13.19)	29	22.90 (14.09)	29	18.20 (13.53)	25	15.65 (14.51)	25	21.10 (16.33)	27	18.20 (16.73)	20	18.66 (14.61)
Modified Coordinate Response Measure														
Threshold	30	−5.15 (5.68)	30	−6.50 (5.66)	29	−6.76 (5.01)	27	−3.88 (4.85)	27	−4.51 (5.66)	27	−4.69 (5.40)	22	−4.05 (4.79)
Self-report														
Glasgow Hearing Aid Benefit Profile														
Initial disability (%)	30	35.00 (15.54)	29	36.42 (15.68)	30	36.88 (15.77)	27	43.29 (16.98)	27	43.98 (17.80)	27	38.66 (20.07)	23	36.84 (22.77)
Handicap (%)	30	29.38 (19.91)	29	29.31 (20.46)	30	24.38 (16.93)	27	35.88 (26.21)	27	34.26 (28.82)	27	30.56 (29.89)	22	30.40 (28.56)
Hearing Handicap Inventory for the Elderly														
Total score	30	34.07 (21.36)	30	30.73 (23.75)	29	28.41 (21.19)	27	40.89 (27.10)	27	39.93 (27.90)	27	37.19 (26.84)	21	35.90 (30.04)

GEE analyses assessed the interaction between performance on untrained tasks as a function of time and treatment group. Results from the GEE analyses are presented in Table 5 and summarized below.

**TABLE 5.** GEE model results for the time (T2–T3, pre-post intervention) by group (active control vs. experimental group) interaction for all participants (n = 57)

Outcomes	T2–T3×Group Interaction		
	Beta (95% CI)	*p*	Cohen’s d (95% CI)
Cognition			
Visual letter monitoring			
Hits, 2s/letter	−0.06 (−1.10 to 0.98)	0.91	0.03 (−0.50 to 0.56)
Hits, 1s/letter	−0.61 (−1.81 to 0.60)	0.32	0.26 (−0.27 to 0.80)
Backward Digit Span			
Trials correct	−1.12 (−2.09 to −0.14)*	0.03*	0.59 (0.05 to 1.13)*
Size Comparison Span			
Total	−0.69 (−2.64 to 1.27)	0.49	0.18 (−0.35 to 0.71)
Intrusions	0.73 (−0.73 to 2.19)	0.33	−0.26 (−0.79 to 0.28)
Dual task listening and memory			
Dual task score	0.515 (−1.78 to 2.81)	0.66	−0.08 (−0.61 to 0.45)
Test of Everyday Attention			
Subtest 6	−0.21 (−0.42 to −0.01)*	0.04*	0.54 (0.00 to 1.08)*
Subtest 7 dual-task decrement	−0.39 (−1.55 to 0.77)	0.51	0.17 (−0.37 to 0.7)
Test of Attention in Listening			
Attend frequency IO	−0.04 (−0.12 to 0.04)	0.37	0.23 (−0.3 to 0.76)
Attend frequency CR	0.02 (−0.05 to 0.09)	0.52	−0.16 (−0.7 to 0.37)
Attend location IO	0.03 (−0.05 to 0.10)	0.49	−0.18 (−0.72 to 0.35)
Attend location CR	−0.02 (−0.10 to 0.06)	0.63	0.13 (−0.41 to 0.66)
Speech perception			
British English Semantic Sentence Test			
High context (%)	4.88 (−3.90 to 13.66)	0.28	−0.38 (−0.92 to 0.15)
Low context (%)	−2.23 (−10.21 to 5.75)	0.58	0.09 (−0.44 to 0.62)
Phoneme discrimination			
Threshold /a/ /e/	−6.64 (−11.97 to −1.31)*	0.02*	0.64 (0.09 to 1.18)*
Threshold /d/ /g/	0.91 (−4.40 to 6.22)	0.74	−0.09 (−0.62 to 0.44)
Modified Coordinate Response Measure			
Threshold	−0.17 (−2.23 to 1.90)	0.88	0.04 (−0.49 to 0.57)
Self-report			
Glasgow Hearing Aid Benefit Profile			
Initial disability (%)	6.16 (0.81 to 11.51)*	0.02	−0.59 (−1.13 to −0.05)*
Handicap (%)	−0.67 (−7.79 to 6.45)	0.85	0.05 (−0.48 to 0.58)
Hearing Handicap Inventory for the Elderly			
Total score	0.97 (−3.75 to 5.70)	0.69	−0.11 (−0.64 to 0.43)

Significance levels are unadjusted for multiple comparisons.

* Statistically significant model results.

##### Cognition •

For the primary outcome measure, the Visual Letter Monitoring (VLM) Task, there was no significant group by time interaction shown in either the 2s/letter or 1s/letter conditions. The results were similar for all other untrained cognitive outcome measures, with the following notable exceptions. For Backward Digit Span (a version of which is trained within Cogmed RM), there was a significant interaction between group and time with a medium effect size (Cohen’s d = 0.59). The TEA subtest 6 also showed a significant interaction with a medium effect size (Cohen’s d = 0.54). However, this significant effect was observed as a result of improved TEA subtest 6 for participants in the Active Control Group, with no improvements shown for participants in the Experimental Group, pre- to post-training (T2 to T3).

##### Speech Perception •

Phoneme discrimination /a/ /e/ was the only speech outcome measure that showed a significant group by time interaction with a medium effect size (Cohen’s d = 0.64). However, closer examination of the group means in Table 4 show that this interaction was driven by the fact that discrimination scores worsened for participants in the Experimental Group T2 to T3 but improved for participants in the Active Control Group.

##### Self-Report •

There was a significant group by time interaction shown for the GHABP Initial Disability scores, with a medium effect size (Cohen’s d = −I.59). This interaction occurred because Initial Disability scores decreased (improved) by 5.3% from T2 to T3 in the Experimental Group, with no change shown for Active Controls.

**Fig. 3.**
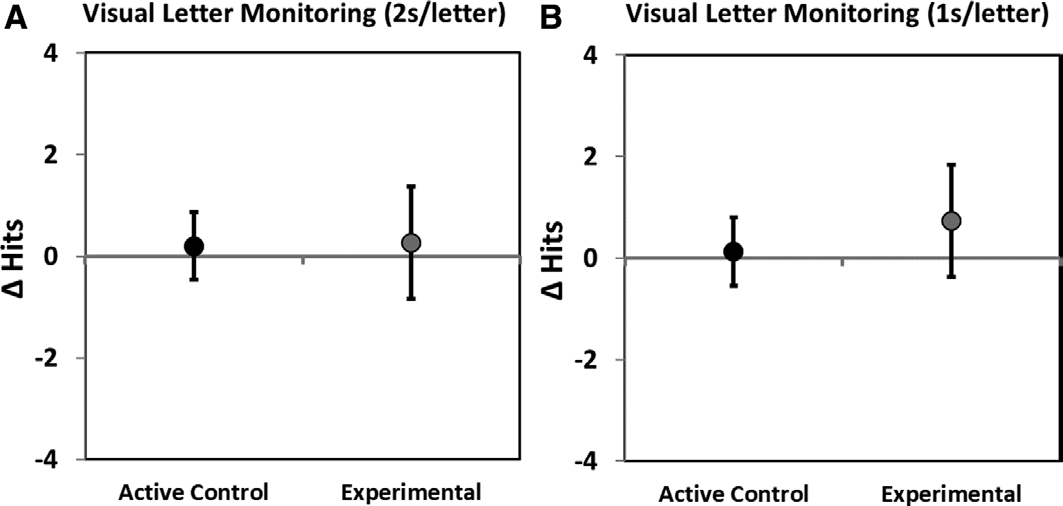
A and B, Pre- to post-training (T2–T3) change in the primary outcome measure Visual Letter Monitoring (A) 2s/letter and (B) 1s/letter for participants in the active control (n = 30) and experimental (n = 27) groups. Improvements in performance are shown as positive values. Error bars = 95% confidence intervals.

Figures 3A, B show no significant change in pre- to post-training performance (unimputed data) for the primary outcome measure (VLM) for either the Experimental or Active Control Groups. Unimputed pre- to post-training change data by group for all included outcome measures are provided in Table 2 in Supplemental Digital Content 1, http://links.lww.com/EANDH/A855.

#### Retention of Learning 6 Months Post-Training (Hypothesis

Table 6 shows post-hoc comparisons within the Experimental Group for the main effects of the intervention period (T2 to T3), and the retention of post-training improvements in outcomes from baseline to 6-month follow-up (T2 to T4).

**TABLE 6.** GEE model results for post-hoc analyses of main effects of intervention (T2–T3) and retention (T2–T4) for participants in the experimental group (n = 27)

Outcomes	Main Effect of Intervention			Retention		
	T2–T3			T2–T4		
	Beta (95% CI)	*p*	Cohen’s d (95% CI)	Beta (95% CI)	*p*	Cohen’s d (95% CI)
Cognition						
Visual letter monitoring						
Hits, 2s/letter	0.26 (−0.67 to 1.19)	0.58	−0.15 (−0.70 to 0.49)	0.58 (−0.53 to 1.69)	0.30	−0.28 (−0.85 to 0.45)
Hits, 1s/letter	0.74 (−0.28 to 1.77)	0.16	−0.39 (−0.89 to 0.35)	0.55 (−0.28 to 1.38)	0.19	−0.35 (−0.81 to 0.31)
Digit span backward						
Trials correct	0.94 (0.22 to 1.67)*	0.01	−0.69 (−1.01 to 0.03)*	1.28 (0.54 to 2.03)*	0.001	−0.92 (−1.18 to −0.12)*
Size Comparison Span						
Total	0.85 (−0.76 to 2.47)	0.30	−0.28 (−0.98 to 0/58)	1.02 (−1.03 to 3.06)	0.33	−0.27 (−1.07 to 0.69)
Intrusions	−0.59 (−1.75 to 0.56)	0.31	0.27 (−0.47 to 0.85)	0.08 (−1.41 to 1.56)	0.92	0.03 (−0.77 to 0.73)
Dual task listening and memory						
Dual task score	1.85 (0.54 to 3.16)*	0.01	−0.75 (−1.24 to 0.17)*	1.72 (−0.53 to 3.97)	0.13	−0.41 (−1.21 to 0.63)
Test of Attention in Listening						
Attend frequency IO	0.02 (−0.04 to 0.07)	0.54	−0.16 (−0.26 to 0.02)	0.05 (−0.02 to 0.12)	0.16	−0.38 (−0.43 to −0.11)
Attend frequency CR	−0.01 (−0.05 to 0.03)	0.64	0.13 (−0.04 to 0.22)	0.03 (−0.02 to 0.08)	0.24	−0.32 (−0.36 to −0.09)
Attend location IO	−0.015 (−0.07 to 0.05)	0.63	0.13 (−0.06 to 0.24)	0.01 (−0.06 to 0.08)	0.78	−0.08 (−0.22 to 0.11)
Attend location CR	0.02 (−0.04 to 0.09)	0.49	−0.19 (−0.29 to 0.03)	0.05 (−0.04 to 0.14)	0.27	0.30 (−0.39 to −0.03)
Test of Everyday Attention						
Subtest 6	−0.019 (−0.19 to 0.15)	0.83	0.06 (−0.21 to 0.29)	−0.19 (−0.35 to −0.03)*	0.02*	0.64 (0.20 to 0.70)*
Subtest 7 dual-task decrement	−0.31 (−0.72 to 0.10)	0.14	0.41 (−0.11 to 0.68)	−0.32 (−1.04 to 0.39)	0.37	0.24 (−0.35 to 0.69)
Speech perception						
Phoneme discrimination						
Threshold /a/ /e/	3.37 (−0.63 to 7.37)	0.10	−0.45 (−1.55 to 0.91)	−1.47 (−4.55 to 1.61)	0.35	0.25 (−0.90 to 1.26)
Threshold /d/ /g/	0.43 (−3.85 to 4.70)	0.85	−0.05 (−1.31 to 1.23)	−3.67 (−9.13 to 1.79)	0.19	0.36 (−1.18 to 1.69)
Modified Coordinate Response Measure						
Threshold	−0.18 (−1.69 to 1.33)	0.82	0.06 (−0.71 to 0.80)	−0.38 (−1.79 to 1.02)	0.59	0.15 (−0.62 to 0.83)
British English Semantic Sentence Test						
High context (%)	0.35 (−5.20 to 5.90)	0.90	−0.03 (−1.47 to 1.42)	−4.45 (−10.45 to 1.56)	0.15	0.39 (−1.22 to 1.79)
Low context (%)	−2.69 (−8.65 to 3.27)	0.38	0.24 (−1.32 to 1.67)	−1.66 (−8.48 to 5.16)	0.63	0.13 (−1.51 to 1.70)
Self-report						
Glasgow Hearing Aid Benefit Profile						
Initial disability (%)	−5.32 (−9.42 to −1.22)*	0.01	0.69 (−0.75 to 1.73)*	−6.13 (−11.17 to −1.09)*	0.02*	0.65 (−0.92 to 1.84)*
Handicap (%)	−3.7 (−9.11 to 1.71)	0.18	0.37 (−1.17 to 1.69)	−3.2 (−8.96 to 2.56)	0.28	0.30 (−1.27 to 1.68)
Hearing Handicap Inventory for the Elderly						
Total score	−2.74 (−6.17 to 0.69)	0.12	0.43 (−0.84 to 1.44)	−4.53 (−9.88 to 0.82)	0.10	0.45 (−1.10 to 1.74)

Significance levels are unadjusted for multiple comparisons.

* Statistically significant model results.

##### Cognition •

For Backward Digit Span, there was a significant within-group main effect of intervention period, with a medium effect size (Cohen’s d = −o.69) and significant retention of post-training improvements with a large effect size (Cohen’s d = −6.92). For the TEA subtest 6, there was no immediate improvement in performance (T2 to T3), but a significant improvement from baseline was detected at the 6-month follow-up assessment (T2 to T4). For the Dual Task, despite a significant improvement for participants in the Experimental Group T2 to T3, this improvement was slightly exceeded by participants in the Active Control Group (see Table 4), hence this did not result in a significant group by time interaction.

##### Speech Perception •

No significant post-hoc results were identified within the Experimental Group for any of the speech perception measures.

##### Self-Report •

For GHABP Initial Disability scores, there was a statistically significant main effect shown for the intervention period (T2 to T3) with a medium effect size (Cohen’s d = 0.69), and this improvement was shown to be retained at the 6-month follow-up (T2 to T4; Cohen’s d = 0.65).

### Secondary Analyses

#### Predicting Post-Training Performance Using Baseline Working Memory (Hypothesis 3)

Results from the linear regression models are shown in Table 7. For on-task learning, individuals’ T2 Backward Digit Span performance account for 27.6% of the variance in Cogmed Index Improvement scores [*F*(1,24) = 9.143, *p* = 0.006; *R^2^* of 0.276].

**TABLE 7.** Simple Regression Models assessing the predictive value of baseline working memory performance on on-task learning and transfer of learning to untrained outcomes, for participants in the experimental group (n = 27)

		Baseline Working Memory Performance (T2 Backward Digit Span)				
	Beta (95% CI)	Standardized Beta	df	*F*	R^2^	*p*
Outcomes						
On-task learning						
Cogmed RM						
Index Improvement	1.89 (0.60 to 3.19)	0.53	1, 24	9.14	0.276	0.006
Transfer to untrained outcomes (ΔT2–T3)						
Cognition*						
Visual Letter Monitoring						
Hits 2s/letter	−0.22 (−0.82 to 0.37)	−0.16	1, 24	0.60	0.025	0.45
Hits 1s/letter	−0.28 (−0.95 to 0.38)	−0.18	1, 23	0.78	0.033	0.39
Size Comparison Span						
Total	0.11 (−0.93 to 1.16)	0.05	1, 24	0.051	0.002	0.82
Intrusions	−0.37 (−1.03 to 0.30)	−0.23	1, 24	1.31	0.052	0.26
Dual task listening and memory						
Dual task score	0.62 (−0.20 to 1.44)	0.31	1, 23	2.43	0.096	0.13
Test of Attention in Listeining						
Attend frequency IO	−0.02 (−0.06 to 0.01)	−0.26	1, 23	1.73	0.070	0.20
Attend frequency CR	−0.00 (−0.03 to 0.03)	−0.05	1,23	0.062	0.003	0.81
Attend location IO	−0.03 (−0.06 to 0.01)	−0.33	1, 23	2.82	0.109	0.11
Attend location CR	−0.02 (−0.07 to 0.02)	−0.24	1, 23	1.38	0.057	0.25
Test of Everyday Attention						
Subtest 6	−0.09 (−0.01 to 0.20)	0.36	1, 24	3.67	0.133	0.07
Subtest 7 dual task decrement	−0.17 (−0.43 to 0.08)	−0.28	1, 24	1.98	0.076	0.17
Speech perception						
Phoneme discrimination						
Threshold /e/ /a/	0.60 (−2.11 to 3.30)	0.10	1, 22	0.21	0.009	0.65
Threshold /d/ /g/	1.32 (−1.62 to 4.26)	0.20	1, 22	0.87	0.038	0.36
British English Semantic Sentence Test						
High context (%)	1.13 (−2.79 to 50.6)	0.13	1, 22	0.36	0.016	0.56
Low context (%)	−1.68 (−5.68 to 2.33)	−0.18	1, 22	0.76	0.033	0.39
Modified Coordinate Response Measure						
Threshold	0.05 (−0.93 to 1.03)	0.02	1, 24	0.01	0.000	0.92
Self-report						
Glasgow Hearing Aid Benefit Profile						
Initial disability (%)	−1.49 (−4.06 to 1.09)	−0.24	1, 24	1.42	0.056	0.25
Handicap (%)	−1.40 (−4.84 to 2.05)	−0.17	1, 24	0.70	0.028	0.41
Hearing Handicap Inventory for the Elderly						
Total score	1.02 (−1.17 to 3.21)	0.19	1, 24	0.92	0.037	0.35

Significance levels are unadjusted for multiple comparisons. Given that baseline Backward Digit Span (T2) is used as a model predictor, this outcome is not included as an outcome measure within the model analyses.

* Statistically significant results.

**Fig. 4.**
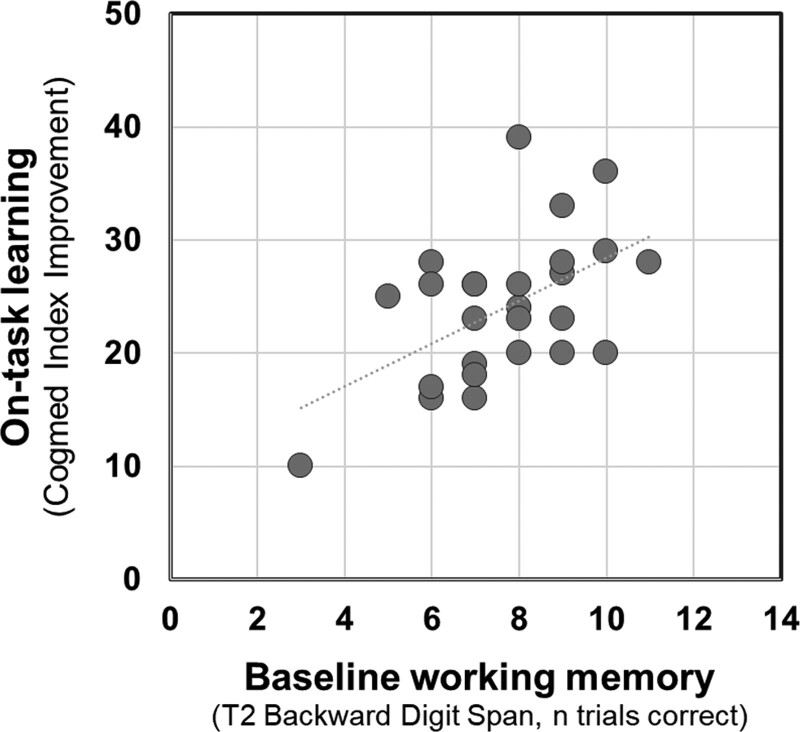
Scatterplot showing baseline working memory (T2 Backward Digit Span, n correct trials) and on-task learning (Cogmed Index Improvement) for participants in the Experimental Group (n = 27).

Figure 4 shows individuals baseline working memory performance (T2 Backward Digit Span) plotted against their on-task learning (Cogmed Index Improvement) for those in the Experimental Group only. The results suggest that for each additional correct trial on the Backward Digit Span task at baseline (T2), participants Cogmed Index Improvement increased by an average of 1.894 points. Baseline working memory was not shown to be predictive of pre- to post-training (T2 to T3) change in performance for any of the untrained outcome measures.

#### Predicting Post-Training Performance Using Baseline Outcome Performance and Degree of On-Task Learning (Hypothesis 4)

The stepwise regression models are shown in Table 3 in Supplemental Digital Content 1, http://links.lww.com/EANDH/A855. For 13/20 of the individual measures, the only reliable predictor of post-training (T3) outcome performance was pretraining (T2) performance on the same measure. This accounted for between 13.7% and 78.7% of variance in post-training performance for cognitive measures [Visual Letter Monitoring 2s/letter Hits 15.5%, 1s/letter Hits 13.7%; Backward Digit Span, 19.2%; Size Comparison Span Total, 63.9%; Dual Task, 63.0%; Test of Everyday Attention Subtest 7 (DTD), 78.7%], between 15.8% and 54.9% for speech perception measures (Phoneme discrimination /e/ /a/, 16.2%, /d/ /g/, 15.8%; British English Semantic Sentence Test High context, 54.9%, Low context, 25.9%; Modified Coordinate Response Measure threshold, 51.3%) and between 68.7% and 88.5% for measures of self-reported hearing, (Glasgow Hearing Aid Benefit Profile Initial Disability, 68.7%, Handicap 75.9%; and Hearing Handicap Inventory for the Elderly total score, 88.5%).

For TAIL attend location IO, and TEA subtest 6, models that predicted post-training (T3) performance using pretraining performance plus degree of on-task learning were statistically superior. Finally, for three outcomes: Size Comparison Span Intrusions, Test of Attention in Listening Attend frequency IO, and Glasgow Hearing Aid Benefit Profile Handicap, models that incorporated the interaction between baseline outcome performance (T2) and degree of on-task learning (Cogmed Index Improvement) offered a significantly improved fit. However, these results should be interpreted with some caution given that the incremental difference in variance accounted for by these models over and above baseline performance alone was very small.

### Quality Assurance of Outcome Measures

Table 4 in Supplemental Digital Content 1, http://links.lww.com/EANDH/A855 shows the results of the intra-class correlations (ICCs), SEM, and MD for all outcome measures across all participants. Following Cicchetti (1994), test-retest reliability scores (reliability coefficients) were interpreted as <0.40 = poor; 0.40 to 0.59 = fair, 0.60 to 0.74 = good, and >0.75 = excellent. Only the GHABP (Initial Disability and Handicap, Hearing Handicap Inventory for the Elderly (total score), and SICSPAN (total) fulfill the criterion of excellent reliability. Backward Digit Span, SICSPAN (intrusions), Dual Task, British English Semantic Sentence Test (High context) and Modified Coordinate Response Measure all demonstrate good reliability. Both Visual Letter Monitoring measures (VLM 2 s/letter and 1 s/letter). Both Phoneme Discrimination measures (threshold /a/ /e/ and threshold /d/ /g/) demonstrate fair reliability, thus results involving these measures should be interpreted with some caution. All four TAIL measures (Attend frequency IO and CR, and Attend location IO and CR) and TEA (subtest 7 DTD) show poor reliability; therefore, results including these measures should be considered unreliable. Finally, it is important to note that pre- to post-training change in outcome performance for participants in the Experimental group (see Table 1 in Supplemental Digital Content 1, http://links.lww.com/EANDH/A855) are consistently smaller than the calculated MDs for all outcomes included in this trial.

## DISCUSSION

Listening to speech-in-noise is a complex and frequent real-world challenge for people with hearing loss and hearing aid users. Impaired auditory function is fundamental to this problem; however, cognitive function also has an important role to play. The current trial sought to examine the benefits of Cogmed training, which primarily targets the storage component of working memory, in an active-controlled RCT of 57 adult hearing aid users and compare findings to those of our prior studies of auditory training that used a similar outcome test battery (Ferguson et al. 2014, Henshaw & Ferguson 2014). In doing so, we sought to generate new knowledge about the processes underpinning Cogmed training and transfer for this population and use this to inform future research directions and targeted intervention developments.

All participants adhered to the full Cogmed training schedule (25/25 sessions, 100% adherence), and analyses showed a highly significant improvement in trained task performance for participants in the Experimental group compared with participants in the Active Control Group who received the same (nonadaptive) training tasks. The only difference between the two training protocols (i.e. the underlying memory process) was the increase in the number of to-be-remembered items (span) for adaptive training in the Experimental group. Results of the GEE analyses showed four statistically significant Time by Group interactions. However, only two of these interactions favoured the Experimental Group over and above the Active Control Group. First, there was a significant interaction shown for a working memory task that was trained within Cogmed, but tested outside of the Cogmed software (Backward Digit Span) in a subtly different way (different talker and presentation software). Second, there was a significant interaction driven by a small improvement in self-reported hearing activity limitations for those in the Experimental Group (as measured by the Initial Disability subscale of the GHABP), for which, despite a statistically significant effect with a medium effect size (Cohen’s d = 0.56), the absolute magnitude of change (<1%) is unlikely to represent anything close to a clinically important effect (Whitmer et al. 2014). The other two significant interactions were shown for the Test of Everyday Attention subtest 6 (whereby participants in the Active Control Group demonstrated improved performance pre- to post-training, with no improvements shown for those in the Experimental Group) and Phoneme discrimination /a/ /e/ thresholds (where performance deteriorated for those in the Experimental Group but improved for participants in the Active Control group T2 to T3, pre- to post-training). Given that the degree of change for significant effects was small and given that there were no corrections for multiple comparisons included in the analyses, it is plausible that the significant effects observed may have arisen by chance. No post-training improvements were shown for working memory measures that were further removed from the trained tasks, including the primary outcome measure (untrained working memory: Visual Letter Monitoring task), nor measures of speech perception performance, or any of the other self-reported outcomes included in the trial.

Examination of individual differences revealed that participants with better baseline working memory performance achieved greater on-task learning (as indicated by a higher Cogmed Index Improvement). Termed the “magnification effect”, this finding has been shown for working memory training interventions in both younger and older adults (Guye et al. 2017; Strobach & Huestegge 2017). However, conflicting evidence suggests the reverse, in that individuals with lower baseline cognition show greater improvements for trained task-switching (Karbach et al. 2017) and working memory (Blacker et al. 2017) tasks (the “compensation’ effect”), as well as greater generalization of learning (Karbach et al. 2017). In the present trial, baseline working memory performance was not shown to be associated with greater generalization of learning to untrained outcomes. Indeed, in the absence of generalized improvements, the only reliable predictor of individuals’ post-training performance on untrained outcome measures was their pretraining performance on those same outcome measures.

Taken together, these results suggest that adaptive Cogmed training is insufficient to elicit domain-general improvements in working memory or far-transfer gains for adult hearing aid users. These findings are consistent with a lack of generalized improvements shown for a crossover study assessing the benefits of Cogmed training within a mixed population of older adults with and without hearing loss (Wayne et al. 2016).

### Mechanisms of Training and Transfer

Cogmed training is designed to challenge and improve components of the working memory system through tasks that are increasingly challenging in terms of the number of to-be-remembered items. Through an adaptive algorithm that is based on trainee performance, the training requires trainees to work at the “edge” or limit of their ability. It is assumed that this will subsequently result in increased working memory capacity (e.g., Klingberg et al. 2002). Yet, as argued by Shipstead et al. (2012), the recall of a greater number of to-be-remembered items in itself does not confirm that working memory capacity has increased. Should Cogmed result in true core training, as evidenced by improved working memory capacity, then we would expect to see improvements for both trained working memory tasks (e.g., backward digit span, simple span) and untrained working memory tasks (e.g., SICspan, complex span). Furthermore, there may also be generalized improvements to other outcomes that rely on working memory (Morrison & Chein 2011). Given that we see no evidence for improvements in untrained tasks of working memory nor related aspects of cognition or speech perception performance in this study, our findings are inconsistent with effective core training. One explanation that has been put forward to explain this is that improvements shown for trained Cogmed tasks may have arisen not from an improvement in working memory capacity itself, but as a result of task-specific abilities (i.e. strategies) that enable improved performance (e.g., Chase & Ericsson 1982; Dunning and Holmes 2014, Harrison et al. 2013, von Bastian & Oberauer 2014). Indeed, following completion of this trial, participants in the Experimental Group were invited to take part in one of two focus groups to discuss their experiences with the working memory training intervention. When asked to describe their training experiences, individuals in the focus groups described using several different strategies to help them deal with the increasing demands of the adaptive training tasks. Their descriptions were consistent with visualization, grouping, chunking, rehearsal, chaining and inhibition. This explanation is in line with a meta-analytic review of published working memory training interventions looking specifically at far-transfer effects, which suggest that working memory training may not genuinely improve working memory capacity but may simply reflect stimulus-specific overlap between trained and transfer tasks, or the development of task-specific strategies (Melby-Lervåg et al. 2016). Should working memory capacity itself be unaffected, then it follows suit that there can be no near- or far-transfer of learning (Shipstead et al. 2012).

There has been considerable debate in the literature as to whether or not Cogmed training improves working memory capacity [see Shipstead et al. (2012) for a review]. Cogmed comprises 11 training tasks that target different components of the working memory system. However, despite being marketed as a verbal and visuospatial working memory training program, the linguistic complexity of training stimuli is capped at digits and letters, and the majority of memory tasks (6/11) train serial order memory storage. Morrison & Chein (2012) argue that if Cogmed training had been consistently shown to improve performance on measures of working memory that assess more than just short-term storage of materials used during training (for example, complex span working memory, change detection and running memory span), then this would provide comprehensive evidence of improved working memory capacity. As it stands, published literature largely supports the statement made by Shipstead et al. (2012) in that “Cogmed will improve performance on tasks that resemble Cogmed training”, and this is almost certainly a direct consequence of the limited working memory processes targeted within training tasks.

Despite some published evidence for Cogmed to result in improved attention, as measured using the Stroop task [see Shipstead et al. (2012) for an overview], as well as improved general attentional stamina (Brehmer et al. 2012, Westerberg & Klingberg 2007), we see no evidence of generalized improvements in attention or focus in the current study for either direct measures of attention, nor for general (untrained) task performance. There are two key aspects to consider here. The first is that the type of task used to assess attention may not be directly aligned with demands on working memory capacity. For example, the Test of Everyday Attention and the Test of Attention in Listening require the effective division of attention (TEA) and response inhibition (TAIL), not working memory per se. A second consideration, which is of particular importance for the design of future research, is that the test-retest reliability of both of these outcome measures is poor. Future training intervention studies should take care to select outcomes based on both the mechanistic alignment of outcomes to trained tasks, as well as outcome reliability, in order to maximise confidence in estimations of effect.

In hindsight, it might be reasonable to suggest that the selection of Cogmed as the core training intervention in this trial was a suboptimal choice, particularly given that our results are inconsistent with effective core working memory training. That is not to say that Cogmed cannot be effective at improving working memory capacity, albeit with refinement to better align training tasks to working memory theory (Gibson et al. 2012; Shipstead et al. 2012). As the fields of hearing research and cognitive hearing science have developed over the last decade, in order to identify effective training approaches to benefit people with hearing loss, it has become clear that we require theory-driven approaches to understand why and how interventions work (e.g., Coulson et al. 2016; Ferguson et al. 2019; Pichora-Fuller et al. 2016). Future directions in training intervention studies for adults with hearing loss should ultimately be directed by the nature of the difficulties people with hearing loss and hearing aid users face. In turn, this will help to clarify the underpinning mechanisms of benefit that should be targeted by the intervention, as well as serve to inform the most appropriate outcomes to assess generalized training-related improvements (Henshaw and Ferguson 2014).

For adults with hearing loss, and even more so for those who use hearing aids, listening is a challenging task that is often made more difficult by competing sounds in the environment (e.g., Lunner et al. 2009). Successful speech-in-noise perception is an everyday goal-directed task that requires active listening. Active listening relies on good executive attention or “cognitive control”, which in turn necessitates a good link between working memory and executive functions (Pichora-Fuller & Phillips 2017). In the initial design and development of this trial, we hypothesized that training a core underpinning cognitive process (working memory capacity) may offer a more direct route to the benefits in working memory, attention, and speech perception that had resulted from our earlier studies of auditory training interventions (Ferguson et al. 2014, Henshaw & Ferguson 2014). Yet, on closer inspection, the results of those initial studies clearly show that the generalized benefits of training are only evident for cognitive and speech tasks that involve working memory updating, attention switching/shifting, or inhibition. There were no improvements shown for tasks that did not tap executive functions (Ferguson & Henshaw 2015). Although these were studies of auditory and not cognitive training, the 3-interval, 3-alternate forced choice paradigm used to deliver the training, required the trainee to simultaneously hold information in memory, while constantly updating that information in order to make same/different comparisons. As such, the transfer of learning shown in these studies may have arisen through the enhancement of cognitive control inherent within an auditory training task that necessitated active listening.

A systematic review and meta-analysis of the efficacy of auditory and cognitive training interventions to improve cognitive performance for adults with hearing loss (Lawrence et al. 2018) showed a small but statistically significant effect of auditory training in terms of generalized improvements in working memory performance, and a large significant effect on overall cognition (combined cognitive tests), with similar results shown for cognitive training. However, a study that used a combined auditory-cognitive approach showed a very large effect (Anderson et al. 2013b), demonstrating that a training task that targets cognitive enhancement delivered using auditory tasks resulted in the largest improvements (by far) in cognitive performance for this population. These results, considered in parallel with our own findings (Ferguson & Henshaw 2015), suggest that combined auditory-cognitive training tasks have the potential to offer superior generalized gains in cognition and speech perception, while maintaining trainee motivation to adhere to training through the use of training tasks with high ecological validity (Henshaw et al. 2015).

### Limitations

There was a greater number of participant withdrawals from the Experimental Group than from the Active Control Group, which was likely in part due to the high demands of the adaptive working memory training tasks, compared with the low-demand nonadaptive version of the training. This was not a problem in the present research as withdrawals did not exceed the anticipated 15% in either group. However, it represents a clear challenge to this type of intervention research and could result in biased group comparisons in future studies if the differences become too pronounced. In a similar vein, despite our best efforts, average active training time was significantly greater for participants in the Experimental Group than the Active Control Group over the 5-week training period. The maximum time-on-task weighting setting offered within the Cogmed software was unable to fully address the imbalance between groups. Additionally, the rigid nature of the training procedure stipulated by Cogmed means that we are unable to identify whether different training durations, or tasks targeting alternative processes, would have resulted in improved training and transfer.

A more pervasive argument is whether nonadaptive training should be used at all as an adaptive control task (Morrison & Chein 2012). The authors argue that more suitable alternatives to active-control tasks for Cogmed studies may include e.g., trivia training (Jaeggi et al. 2011), or adaptive visual search (Redick et al. 2012). Yet, a critical component of using nonadaptive training as an active control task is that it helps to control for participant expectations of improvement by enabling effective blinding of allocation to experimental and control groups. Failure to do so in studies of psychological interventions (e.g., by employing “no-contact” control groups, or tasks that may have differential expectations of improvement) is argued to be “not a minor omission–it is a fundamental design flaw that potentially undermines any causal inference” (Boot et al. 2013, p.445). A nonadaptive version of the same task used in the experimental condition enables the interface, the task and the allocation to be identical for participants in each group. Yet, for all of the reasons outlined above, there are several ways beyond allocation in which the tasks are not identical (e.g., the level of challenge, and time-on-task), which could result in differential expectations and demand characteristics between experimental and active-control groups. Boot et al. (2013) argue that as a way forward, it would be important to measure and account for the influence of any differential expectations of improvements between groups, and by carefully selecting outcome measures that are not readily influenced by differential expectations (Boot et al. 2013).

Although adherence to training was 100% for participants in both groups, it is important to consider the role of the “training aides” (a recommendation of Pearson, the providers of Cogmed training), as well as the active progress monitoring offered by the research team. It is not known what would have happened to training adherence in the absence of such support.

Finally, it is important to acknowledge that the method of scoring for cognitive tasks can have important consequences for task performance. Within this study, we employed a discontinuation rule for the Backward Digit Span task whereby the task is terminated if a participant provides an incorrect response for both trials at a given digit list length. Future studies might consider scoring approaches that exhaust all available performance information rather than adopting termination rules that could disadvantage participants who happen to fail at both instances of a list length, particularly if this occurs early in the task (see Conway et al. 2005).

## CONCLUSIONS

We found no reliable evidence for the generalization of on-task learning to improvements in untrained objective measures of cognition or speech perception for adult hearing aid users within a blinded, active-controlled RCT of Cogmed RM training.

For participants in the Experimental Group who received adaptive training, there was a statistically significant improvement shown for a trained working memory task assessed outside of the working memory training program (Backward Digit Span), and a statistically significant improvement in self-reported hearing ability (Glasgow Hearing Aid Benefit Profile). Both of these effects remained at a 6-months post-training follow-up assessment. However, despite moderate effect sizes, the overall magnitude of the effects rendered the clinical importance of the improvements low. It is hypothesized that improvements for trained tasks may be more representative of task-based strategy development than improvements in working memory capacity.

Future research should assess the benefits of training interventions that target the enhancement of cognition in the context in which it is employed within everyday communication, such as more dynamic aspects of cognitive control. Published evidence suggests that for people with hearing loss, the greatest benefits may be achieved via interventions that combine both auditory and cognitive training elements, particularly those that target the improvement of executive functions using auditory-based training tasks. Results from our quality assurance analyses show that for the selection of outcome measures for future training studies, it is important to consider not only the theoretical concepts of mechanisms of benefit and transfer, but also to select the most reliable measures for each outcome domain assessed. Finally, training should use stimuli that are relevant to the everyday listening challenges of people with hearing loss (e.g., speech) so that trainees are motivated to engage and adhere.

## ACKNOWLEDGMENTS

This article reports independent research supported by the National Institute for Health Research (NIHR) Biomedical Research Unit Funding Programme (BRU-2011-20040) and NIHR Research for Patient Benefit Programme (PB-PG-0816-20044). The views expressed in this article are those of the author(s) and not necessarily those of the NHS, the NIHR, or the Department of Health and Social Care.

We thank Michael LeBeau and Sandra Smith for assistance with participant recruitment, data collection and data management. Thanks to Mr Rajnikant Mehta for the data analysis plan. Thank you to Mr Peter Watson, Dr Duncan Astle and Prof Susan Gathercole, Mr Oliver Zobay and Dr Anne Olson for their expert advice and insight during design, conduct, analysis, and/or write-up of this research.

## Supplementary Material


