# Supplementary material for: Cogmed Training Does Not Generalize to Real-World Benefits for Adult Hearing Aid Users: Results of a Blinded, Active-Controlled Randomized Trial
Source: Ear Hear. 2021 Sep 14;43(3):741–63. doi: 10.1097/AUD.0000000000001096 (PMC9007089; doi:10.1097/AUD.0000000000001096)
Supplement: Supplementary file 1 [file aud-43-0741-s001.pdf]

Supplemental Table S1: Sensitivity analysis for GEE that used multiple imputations to account for missing data. Only outcome variables for which missing data occur are reported. Shaded rows represent results reported in the primary analyses in Table 4.

	**T2-T3*Group Interaction**		
**Outcomes (n missing data)**	**Beta (95% CI)**	**p**	**Cohen's d (95% CI)**
**Cognition**			
**Visual Letter Monitoring (VLM)**			
**Hits, 1s/letter (n=1 missing)**			
Imputed with boundary min (0)	-0.42 (-1.67, 0.83)	0.51	0.18 (-0.36, 0.71)
Imputed with boundary max (10)	-0.79 (-2.06, 0.47	0.22	0.33 (-0.21, 0.86)
Imputed with individual mean (5)	-0.61 (-1.81, 0.60)	0.32	0.26 (-0.27, 0.8 0)
**Test of Everyday Attention (TEA)**			
**Subtest 7 dual task decrement (DTD) (n=1 missing)**			
Imputed with boundary min (-1.2)	-0.46 (-1.62, 0.70)	0.44	0.19 (-0.34, 0.73)
Imputed with boundary max (4.3)	-0.28 (-1.47, 0.91)	0.65	0.11 (-0.42, 0.65)
Imputed with individual mean (0.8)	-0.39 (-1.55, 0.77)	0.51	0.17 (-0.37, 0.70)
**Test of Attention in Listening (TAIL)**			
**Attend frequency IO (n=1 missing)**			
Imputed with boundary min (-0.37)	-0.02 (-0.11, 0.06)	0.60	0.14 (-0.40, 0.67)
Imputed with boundary max (0.28)	-0.05 (-0.13, 0.04)	0.26	0.29 (-0.25, 0.82)
Imputed with individual mean (0)	-0.04 (-0.12, 0.04)	0.37	0.23 (-0.30, 0.76)
**Attend frequency CR (n=2 missing)**			
Imputed with boundary min (-0.28, -0.28)	0.036 (-0.04, 0.11)	0.33	-0.25 (-0.79, 0.28)
Imputed with boundary max (0.44, 0.44)	0.018 (-0.02, 0.06)	0.72	0.09 (-0.44, 0.62)
Imputed with T1 and individual mean (-0.21, -0.01)	0.022 (-0.05, 0.09)	0.52	-0.16 (-0.70,0.37)
MICE	0.020 (-0.04, 0.09)	0.54	-0.16 (-0.70, 0.37)
**Attend location IO (n=1 missing)**			
Imputed with boundary min (-0.21)	0.03 (-0.04, 0.11)	0.37	-0.24 (-0.77, 0.30)
Imputed with boundary max (0.38)	0.01 (-0.07, 0.09)	0.77	-0.08 (-0.61, 0.45)
Imputed with individual mean (0)	0.03 (-0.05, 0.10)	0.49	-0.18 (-0.72, 0.35)
**Attend location CR (n=1 missing)**			
Imputed with boundary min (-0.07)	-0.02 (-0.10, 0.06)	0.66	0.12 (-0.42, 0.65)
Imputed with boundary max (0.49)	-0.04 (-0.12, 0.05)	0.38	0.24 (-0.3, 0.77)
Imputed with individual mean (-0.03)	-0.02 (-0.10, 0.06)	0.63	0.13 (-0.41, 0.66)
**Speech perception**			
**Phoneme discrimination**			
**Threshold /a/ /e/ (n=2 missing)**			
Imputed with boundary min (55, 55)	**-7.75 (-13.06, -2.44)**	**0.004**	**0.75 (0.20, 1.30)**
Imputed with boundary max (98, 98)	-4.56 (-10.95, 1.83)	0.16	0.37 (-0.16, 0.91)
Imputed with individuals' T1 scores (69, 71)	**-6.64 (-11.97, -1.31)**	**0.02**	**0.64 (0.09, 1.18)**
Mice (m = 5)	**-6.96 (-12.27, -1.65)**	**0.01**	**0.64 (0.09, 1.18)**
**threshold /d/ /g/ (n=2 missing)**			
Imputed with boundary min (60,60)	-1.796 (-8.30, 4.71)	0.59	0.14 (-0.39, 0.68)
Imputed with boundary max (99,99)	1.09 (-4.20, 6.39)	0.69	-0.11 (-0.64, 0.43)
Imputed with individuals' T1 scores (99, 94)	0.91 (-4.40, 6.22)	0.74	-0.09 (-0.62, 0.44)
Mice (m=5)	0.72 (-4.65, 6.09)	0.79	-0.09 (-0.62, 0.44)
**Modified Coordinate Response Measure (MCRM)**			
**Threshold (n=1 missing)**			
Imputed with boundary min (-16.5)	-0.40 (-2.52, 1.71)	0.71	0.10 (-0.43, 0.63)
Imputed with boundary max (7.33)	0.39 (-1.94, 2.72)	0.74	-0.08 (-0.62, 0.45)
Imputed with individual mean (-9.33)	-0.17 (-2.23, 1.90)	0.88	0.04 (-0.49, 0.57)

Self-report			
**Glasgow Hearing Aid Benefit Profile (GHABP)**			
**Initial disability % (n=1 missing)**			
Imputed with boundary min (12.5)	**6.57 (1.10, 12.04)**	**0.02**	**-0.62 (-1.16, -0.07)**
Imputed with boundary max (81.25)	4.28 (-1.97, 10.53)	0.18	-0.35 (-0.88, 0.19)
Imputed with individuals' T1 score (25)	**6.16 (0.81, 11.51)**	**0.02**	**-0.59 (-1.13, -0.05)**
**Handicap % (n=1 missing)**			
Imputed with boundary min (0)	-0.26 (-7.41, 6.90)	0.94	0.02 (-0.51, 0.55)
Imputed with boundary max (100)	-3.59 (-12.74, 5.56)	0.44	0.20 (-0.34, 0.73)
Imputed with individual mean (12.5)	-0.67 (-7.79, 6.45)	0.85	0.05 (-0.48, 0.58)
**Hearing Handicap Inventory for the Elderly (HHIE)**			
**Total score (n=1 missing)**			
Imputed with boundary min (2)	-0.46 (-5.26, 4.34)	0.85	0.05 (-0.48, 0.58)
Imputed with boundary max (100)	2.81 (-3.80, 9.42)	0.40	-0.21 (-0.74, 0.32)
Imputed with individual mean (45)	0.97 (-3.75, 5.70)	0.69	-0.11 (-0.64, 0.43)

Statistically significant results are indicated in **bold**. Significance levels are unadjusted for multiple comparisons.

Supplemental Table S2: Change in outcome performance for untrained measures of cognition, speech perception and self-reported hearing for participants in the Active Control (n=30) and Experimental (n-27) Groups.

Outcomes	Within-group comparisons (Δ T2-T3)		Between-group comparisons				
	**Active Control (n=30)**	**Experimental (n=27)**					
	**Mean difference (SD; 95% CI)**	**Mean difference (SD; 95% CI)**	**Mean difference (SE; 95% CI)**	**df**	**t**	**p**	**Cohen's d (95% CI)**
**Cognition**							
**Visual Letter Monitoring (VLM)**							
Hits, 2s/letter	0.20 (1.32; -0.29, 0.69)	0.26 (2.51; -0.73, 1.25)	0.06 (0.52; -0.99, 1.11)	55	.113	0.910	0.03 (-0.47, 0.53)
Hits, 1s/letter	0.13 (1.81; -0.54, 0.81)	0.73 (2.82; -0.41, 1.87)	0.60 (0.63; -0.66, 1.85)	54	.955	0.344	0.26 (-0.34, 0.86)
**Backward Digit Span**							
Trials correct	-0.17 (1.82; -0.85, 0.51)	**0.96 (2.01; 0.11, 1.81)***	**1.13 (0.52; 0.08, 2.17)**	**52**	**2.154**	**0.036**	**0.60 (0.10, 1.10)**
**Size Comparison Span (SICSPAN)**							
Total	0.17 (3.13; -1.00, 1.34)	0.85 (4.36; -0.87, 2.58)	0.69 (1.00; -1,31, 2.68)	55	.687	0.495	0.19 (-0.77, 1.14)
Intrusions□	-0.13 (2.54; -1.08, 0.82)	0.59 (3.12; -0.64, 1.83)	0.73 (0.75; -2.23, 0.75)	55	.968	0.338	0.26 (-0.46, 0.98)
**Dual task listening and memory**							
Dual task score	**2.54 (5.12; 0.55, 4.52)***	**1.96 (3.56; 0.52, 3.40)***	-0.57 (1.21; -3.00, 1.85)	52	-.475	0.637	-0.13 (-1.29, 1.03)
**Test of Everyday Attention (TEA)**							
Subtest 6□	**0.23 (0.34;0.11, 0.36)***	-0.02 (0.45; -0.20, 0.16)	**-0.21 (0.11; -0.43, -0.00)**	**55**	**-2.038**	**0.046**	**-0.55 (-0.65, -0.45)**
Subtest 7 dual task decrement (DTD)□	0.73 (3.13; -0.46, 1.92)	0.31 (1.11; -0.13, 0.75)	-0.42 (0.64; -1.70, 0.86)	54	-0.659	0.513	-0.18 (-0.79, 0.43)
**Test of Attention in Listening (TAIL)**							
Attend frequency IO□	0.02 (0.17; -0.05, 0.08)	-0.02 (0.15; -0.08, 0.04)	-0.04 (0.04; -0.12, 0.05)	54	-.880	0.383	-0.24 (-0.28, -0.20)
Attend frequency CR□	-0.01 (0.15; -0.07, 0.05)	0.01 (0.12; -0.04, 0.06)	0.02 (0.04; -0.05, 0.09)	53	-.517	0.607	0.14 (0.11, 0.18)
Attend location IO□	-0.01 (0.13; -0.06, 0.04)	0.02 (0.16; -0.05, 0.09)	0.03 (0.04; -0.05, 0.11)	54	.757	0.452	0.21 (0.17, 0.24)
Attend location CR□	-0.00 (0.11; -0.05, 0.04)	-0.03 (0.18; -0.10, 0.05)	-0.02 (0.04; -0.10, 0.06)	54	-.585	0.561	0.21 (0.17, 0.25)
**Speech perception**							
**Phoneme discrimination**							
Threshold /a/ /e/□	3.27 (9.97; -6.99, 0.46)	-4.00 (10.90; -0.50, 8.50)	**-7.27 (2.82; -12.92, -1.62)**	**53**	**-2.579**	**0.013**	**-0.71 (-3.41, 1.99)**
Threshold /d/ /g/□	-1.33 (8.95; -2.01, 4.67)	-0.30 (11.98; -4.65, 5.25)	1.03 (2.82; -4.63, 6.70)	53	.366	0.716	0.10 (-2,61, 2.81)
**British English Semantic Sentence Test (BESST)**							
High context %	5.84 (18.38; -1.29, 12.96)	0.38 (15.61; -6.07, 6.82)	-5.46 (4.71; -14.93, 4.01)	51	-1.158	0.252	-0.33 (-4.85, 4.20)
Low context %	-3.90 (15.34; -9.85, 2.05)	-1.81 (15.96; -8.40, 4.77)	2.08 (4.30; -6.55, 10.72)	51	.484	0.630	0.14 (-3.99, 4.27)
**Modified Coordinate Response Measure (MCRM)**							
Threshold□	0.35 (4.08; -1.21, 1.90)	0.18 (4.08; -1.43, 1.79)	-0.17 (1.09; -2.35, 2.02)	54	-.152	0.880	-0.04 (-1.09, 1.01)

**Self-report**							
**Glasgow Hearing Aid Benefit Profile (GHABP)**							
Initial disability %□	-0.65 (9.93; -4.42, 3.13)	**5.32 (11.06; 0.95, 9.70)***	**5.97 (2.81; 0.35, 11.59)**	**54**	**2.128**	**0.038**	**0.58 (-2.12, 3.28)**
Handicap %□	4.31 (13.37; -0.78, 9.40)	3.70 (14.63; -2.08, 9.49)	-0.61 (3.74; -8.11, 6.90)	54	-.162	0.872	-0.04 (-3.64, 3.56)
**Hearing Handicap Inventory for the Elderly (HHIE)**							
Total score□	2.41 (8.66; -0.88, 5.71)	2.74 (9.28; -0.93, 6.41)	0.33 (2.40; -4.48, 5.13)	54	.136	0.892	0.04 (-2.27, 2.34)

□Variables for which lower scores indicate better performance are reversed in the calculation of pre- to post-training change so that improvements in performance are always shown as positive values in this table. Statistically significant results are indicated in **bold**. *p<.05 for within-group comparisons. Significance levels are unadjusted for multiple comparisons.

***Supplemental Table S3*: General Linear Models (GLMs) assessing the predictive value of pre-training outcome performance, degree of on-task learning or their interaction on post-training outcome performance, for participants in the Experimental Group (n=27).**

Outcome model	R^2	Adj. R^2	*df*	*ΔF*	*p*
**Cognition**					
**Visual Letter Monitoring Hits 2s/letter, post-training (T3)**					
**Step 1: pre-training (T2)**	**.187**	**.155**	**1,25**	**5.77**	**.02**
Step 2: pre-training (T2), on-task learning	.189	.121	1,24	.04	.85
Step 3: pre-training (T2), on-task learning, interaction	.226	.125	1,23	1.12	.30
**Visual Letter Monitoring Hits 1s/letter, post-training (T3)**					
**Step 1: pre-training T2**	**.171**	**.137**	**1.24**	**4.96**	**.04**
Step 2: pre-training (T2), on-task learning	.172	.100	1.23	.02	.88
Step 3: pre-training (T2), on-task learning, interaction	.196	.086	1,22	.65	.43
**Backward Digit Span, post-training (T3)**					
**Step 1: pre-training (T2)**	**.227**	**.192**	**1,22**	**6.48**	**.02**
Step 2: pre-training (T2), on-task learning	.278	.209	1,21	1.47	.24
Step 3: pre-training (T2), on-task learning, interaction	.278	.170	1,20	.01	.92
**SICSPAN Total, post-training (T3)**					
**Step 1: pre-training (T2)**	**.653**	**.639**	**1,25**	**47.07**	**.000**
Step 2: pre-training (T2), on-task learning	.657	.628	1,24	.28	.60
Step 3: pre-training (T2), on-task learning, interaction	.658	.613	1,23	.06	.80
**SICSPAN Intrusions, post-training (T3)**					
**Step 1: pre-training (T2)**	**.497**	**.477**	**1,25**	**24.70**	**.000**
Step 2: pre-training (T2), on-task learning	.537	.499	1,24	2.09	.16
**Step 3: pre-training (T2), on-task learning, interaction**	**.689**	**.649**	**1,23**	**11.27**	**.003**
**Dual Task listening and memory, post-training (T3)**					
**Step 1: pre-training (T2)**	**.645**	**.630**	**1,24**	**43.54**	**.000**
Step 2: pre-training (T2), on-task learning	.670	.641	1,23	1.78	.20
Step 3: pre-training (T2), on-task learning, interaction	.671	.627	1,22	.09	.77
**TAIL Attend frequency IO, post-training (T3)**					
**Step 1: pre-training (T2)**	**.186**	**.152**	**1,24**	**5.49**	**.03**
Step 2: pre-training (T2), on-task learning	.192	.121	1,23	.16	.69
**Step 3: pre-training (T2), on-task learning, interaction**	**.358**	**.270**	**1,22**	**5.69**	**.03**
**TAIL Attend frequency CR, post-training (T3)**					
Step 1: pre-training T2	.149	.113	1,24	4.20	.05
Step 2: pre-training (T2), on-task learning	.151	.078	1,23	.07	.80
Step 3: pre-training (T2), on-task learning, interaction	.209	.101	1,22	1.61	.22
**TAIL Attend location IO, post-training (T3)**					
Step 1: pre-training (T2)	.053	.013	1.24	1.34	.26
**Step 2: pre-training (T2), on-task learning**	**.253**	**.188**	**1,23**	**6.143**	**.02**
Step 3: pre-training (T2), on-task learning, interaction	.253	.151	1,22	.001	.98
**TAIL Attend location CR, post-training (T3)**					
Step 1: pre-training (T2)	.001	-.040	1,24	.029	.87
Step 2: pre-training (T2), on-task learning	.004	-.082	1,23	.072	.79
Step 3: pre-training (T2), on-task learning, interaction	.005	-.131	1,22	.015	.90
**TEA Subtest 6 post-training (T3)**					
**Step 1: pre-training (T2)**	**.564**	**.546**	**1.25**	**32.29**	**.000**
**Step 2: pre-training (T2), on-task learning**	**.694**	**.669**	**124**	**10.28**	**.004**
Step 3: pre-training (T2), on-task learning, interaction	.697	.658	1.23	.22	.65
**TEA Subtest 7 dual task decrement (DTD), post-training (T3)**					
**Step 1: pre-training (T2)**	**.795**	**.787**	**1,25**	**97.20**	**.000**
Step 2: pre-training (T2), on-task learning	.806	.790	1.24	1.36	.26
Step 3: pre-training (T2), on-task learning, interaction	.808	.783	1.23	.15	.71
**Speech perception**					

**Phoneme discrimination threshold /e/ /a/, post-training (T3)**					
**Step 1: pre-training (T2)**	**.197**	**.162**	**1,23**	**5.63**	**.03**
Step 2: pre-training (T2), on-task learning	.199	.126	1,22	.06	.82
Step 3: pre-training (T2), on-task learning, interaction	.202	.088	1,21	.10	.76
**Phoneme discrimination threshold /d/ /g/, post-training (T3)**					
**Step 1: pre-training (T2)**	**.193**	**.158**	**1,23**	**5.50**	**.03**
Step 2: pre-training (T2), on-task learning	.200	.127	1,22	.19	.67
Step 3: pre-training (T2), on-task learning, interaction	.206	.093	1,21	.17	.69
**British English Semantic Sentence Test (BESST) High Context %, post-training (T3)**					
**Step 1: pre-training (T2)**	**.568**	**.549**	**1,23**	**30.27**	**.000**
Step 2: pre-training (T2), on-task learning	.570	.531	1,22	.10	.76
Step 3: pre-training (T2), on-task learning, interaction	.579	.519	1,21	.46	.51
**British English Semantic Sentence Test (BESST) Low Context %, post-training (T3)**					
**Step 1: pre-training (T2)**	**.290**	**.259**	**1,23**	**9.39**	**.005**
Step 2: pre-training (T2), on-task learning	.290	.225	1,22	.00	.97
Step 3: pre-training (T2), on-task learning, interaction	.293	.192	1,21	.10	.76
**Modified Coordinate Response Measure (MCRM) Threshold, post-training (T3)**					
**Step 1: pre-training (T2)**	**.532**	**.513**	**1,25**	**28.41**	**.000**
Step 2: pre-training (T2), on-task learning	.534	.495	1,24	.08	.78
Step 3: pre-training (T2), on-task learning, interaction	.558	.501	1,23	1.30	.266
**Self-report**					
**Glasgow Hearing Aid Benefit Profile (GHABP) Initial Disability, post-training (T3)**					
**Step 1: pre-training (T2)**	**.699**	**.687**	**1,25**	**58.04**	**.000**
Step 2: pre-training (T2), on-task learning	.713	.689	1,24	1.14	.30
Step 3: pre-training (T2), on-task learning, interaction	.713	.676	1,23	.04	.85
**Glasgow Hearing Aid Benefit Profile (GHABP) Handicap, post-training (T3)**					
**Step 1: pre-training (T2)**	**.768**	**.759**	**1,25**	**82.78**	**.000**
Step 2: pre-training (T2), on-task learning	.791	.773	1,24	2.59	.12
**Step 3: pre-training (T2), on-task learning, interaction**	**.831**	**.809**	**1,23**	**5.44**	**.03**
**Hearing Handicap Inventory for the Elderly (HHIE) Total score, post-training (T3)**					
**Step 1: pre-training (T2)**	**.890**	**.885**	**1,25**	**201.46**	**.000**
Step 2: pre-training (T2), on-task learning	.896	.887	1,24	1.42	.25
Step 3: pre-training (T2), on-task learning, interaction	.909	.897	1,23	3.25	.08

Statistically significant results are indicated in **bold**. Significance levels are unadjusted for multiple comparisons.

Supplemental Table S4: Quality assurance analyses including retest reliability (T1-T2) intra-class correlations (ICCs), Standard Error of Measurement (SEM), and Minimal Difference (MD) for all outcome measures across all participants (n-57).

Outcome	n	$ICC_{2,1}$	$ICC_{3,1}$	SEM	MD
**Cognition**					
**Visual Letter monitoring (VLM)**					
Hits, 2s/letter	57		0.43	1.33	3.68
Hits, 1s/letter	57		0.58	1.83	5.07
**Backward Digit Span**					
Trials correct	56	0.72		1.62	4.48
**Size Comparison Span (SICSPAN)**					
Total	57	0.78		4.48	12.41
Intrusions	57		0.66	2.30	6.39
**Dual task listening and memory**					
Dual task score	57	0.67		3.97	10.99
**Test of Everyday Attention(TEA)**					
Subtest 6	56	0.75		0.39	1.08
Subtest 7 dual task decrement (DTD)	56		0.38	1.39	3.86
**Test of everyday Listening (TAIL)**					
Attend frequency IO	56		-0.05	0.00	0.00
Attend frequency CR	55		0.09	0.04	0.12
Attend location IO	56		0.12	0.06	0.16
Attend location CR	56		0.21	0.07	0.20
**Speech perception**					
**Phoneme discrimination**					
Threshold /a/ /e/	54	0.46		7.98	22.11
Threshold /d/ /g/	54		0.57	8.10	22.45
**British English Semantic Sentence Test (BESST)**					
High context %	52	0.65		15.10	41.87
Low context %	52	0.35		10.18	28.21
**Modified Coordinate Response Measure (MCRM)**					
Threshold	57	0.74		3.41	9.46
**Self-report**					
**Glasgow Hearing Aid Benefit Profile (GHABP)**					
Initial disability %	56		0.84	8.65	23.98
Handicap %	56		0.83	12.67	35.13
**Hearing Handicap Inventory for the Elderly (HHIE)**					
Total score	57	0.92		9.58	26.57
